# Myo1b promotes tumor progression and angiogenesis by inhibiting autophagic degradation of HIF-1α in colorectal cancer

**DOI:** 10.1038/s41419-022-05397-1

**Published:** 2022-11-08

**Authors:** Yi-Hong Chen, Nan-Zhu Xu, Chang Hong, Wen-Qi Li, Yi-Qiong Zhang, Xin-Yi Yu, Yue-Le Huang, Jue-Yu Zhou

**Affiliations:** 1 Department of Biochemistry and Molecular Biology, School of Basic Medical Sciences, Southern Medical University, Guangzhou, 510515 P.R. China; 2 The First School of Clinical Medicine, Southern Medical University, Guangzhou, 510515 P.R. China

**Keywords:** Prognostic markers, Tumour angiogenesis, Colorectal cancer

## Abstract

Myosin 1b (Myo1b) is an important single-headed membrane-associated motor of class I myosins that participate in many critical physiological and pathological processes. Mounting evidence suggests that the dysregulation of Myo1b expression has been extensively investigated in the development and progression of several tumors. However, the functional mechanism of Myo1b in CRC angiogenesis and autophagy progression remains unclear. Herein, we found that the expression of Myo1b was upregulated in CRC tissues and its high expression was correlated with worse survival. The overexpression of Myo1b promoted the proliferation, migration and invasion of CRC cells. Conversely, silencing of Myo1b suppressed tumor progression both in vitro and in vivo. Further studies indicated that Myo1b inhibited the autophagosome-lysosome fusion and potentiated the VEGF secretion of CRC cells to promote angiogenesis. Mechanistically, Myo1b blocked the autophagic degradation of HIF-1α and then led to the accumulation of HIF-1α, thus enhancing VEGF secretion and then promoting tumor angiogenesis in CRC. Together, our study provided novel insights into the role of Myo1b in CRC progression and revealed that it might be a feasible predictive biomarker and promising therapeutic target for CRC patients.

## Introduction

Colorectal cancer (CRC) is the third most common cancer and the second leading cause for cancer-related death worldwide and its incidence is rising rapidly in young and middle-aged adults [1, 2]. In spite of the standard treatment including surgery, radiation, chemotherapy, there is still no valid and reliable means to improve the survival rate of CRC patients in advanced stages [3]. Moreover, tumor-induced angiogenesis is a key hallmark of carcinogenesis, which is also responsible for cancer cell growth and metastasis in CRC, and thus targeted antiangiogenic therapies have been a promising option for CRC treatment [4].

Myosins represent a large family of actin motor proteins that play key roles in regulating actin cytoskeleton architecture and dynamics [5]. Emerging evidence has revealed that myosins function as essential regulators of tumorigenesis, playing either oncogenic or tumor-suppressing roles [6, 7]. Myo1b, an important member of class I myosins, is a single-headed membrane-associated motor that binds to actin filaments with a catch-bond behavior in response to load and participates in many critical physiological and pathological processes. Recently, aberrant expression of Myo1b has been detected in various cancers, including prostate cancer [6], head and neck squamous cell carcinoma (HNSCC) [8–10], cervical cancer [11, 12], esophageal squamous cell carcinoma (ESCC) [13] and glioma [14]. Ohmura et al. demonstrated that silencing Myo1b suppressed the migration and invasion of HNSCC cells by inhibiting large protrusion formation of cell membranes [10]. Furthermore, miR-363 and miR-145-3p were found to be a tumor-suppressor in HNSCC or ESCC via targeting Myo1b [8, 9, 13]. Our previous study suggested a potential role of Myo1b in cervical carcinogenesis and its over-expression was significantly correlated with FIGO Stage, HPV infection, lymph node metastasis and pathological grade [12]. Although a recent report showed that Myo1b is a pro-invasive regulator marker of membrane protrusion in CRC [15], the studies of Myo1b on CRC tumorigenesis are still limited, and their functional mechanisms remain poorly understood.

Hypoxia inducible factor-1α (HIF-1α) and vascular endothelial growth factor (VEGF) are two key angiogenic regulatory factors. Several genes have been proven to play crucial roles in tumor angiogenesis by directly acting on VEGF or stimulating angiogenesis through HIF-1α/VEGF pathway. For instance, ALDH1A1 has been shown to promote tumor angiogenesis via retinoic acid/HIF-1α/VEGF signaling in MCF-7 breast cancer cells [16]. However, whether Myo1b controls the CRC angiogenesis also has not been explored.

Herein, we investigated the expression levels of Myo1b in CRC tissues compared with matched non-tumor tissues and demonstrated the pivotal function of Myo1b in CRC progression and angiogenesis. Functional studies indicated that Myo1b acts as a pivotal autophagy regulator by modulating the autophagosome-lysosome fusion, which could inhibit the autophagic degradation of HIF-1α, thus enhancing VEGF secretion and then promoting tumor angiogenesis in CRC. Together, these data provided novel insights into the role of Myo1b in CRC progression and revealed that it might be a feasible predictive biomarker and promising therapeutic target for CRC patients.

## Results

### Myo1b expression is increased in CRC and associated with the malignant phenotypes

**Fig. 1:**
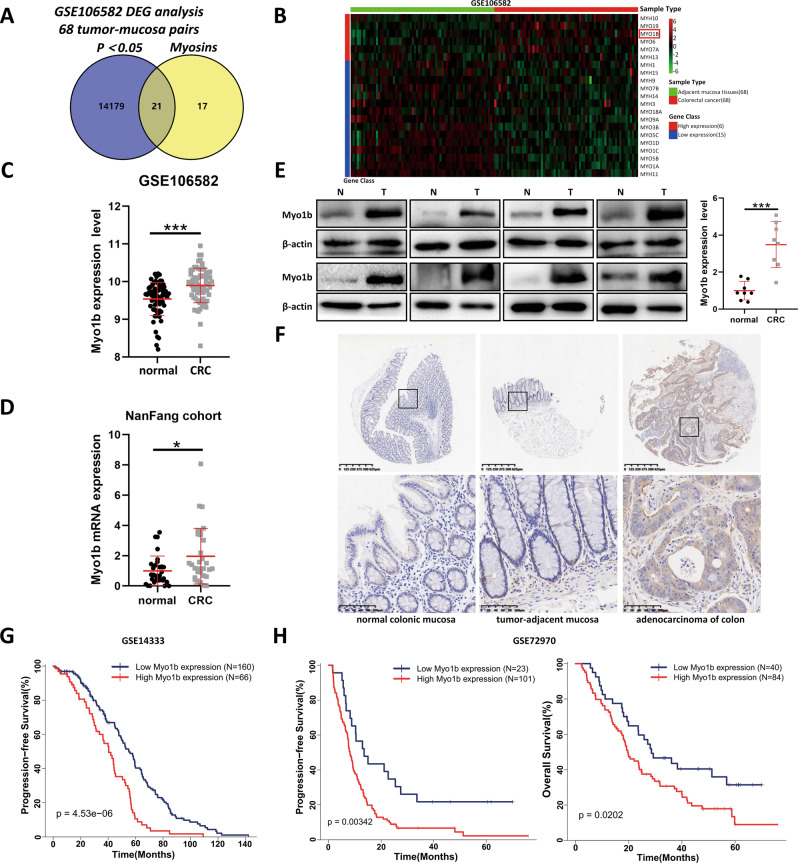
Myo1b is upregulated in colorectal cancer and associated with poor prognosis. **A** Venn diagram depicting the overlap of differential expressed genes (DEGs) between 68 pairs CRC with adjacent mucosa tissues in the GEO dataset (GSE1065862) and myosin family genes. **B** Heatmap enumerates the differential expressed genes encoding Myosins between adjacent mucosa tissues and colorectal cancer samples in the GEO dataset (GSE106582). **C** Scatter diagram represents quantification analysis of Myo1b expression in adjacent mucosa tissues (*n* = 68) and colorectal cancer (*n* = 68) from the GEO dataset (GSE106582). **D** Scatter diagram represents Myo1b mRNA expression level of adjacent mucosa tissues (*n* = 31) and colorectal cancer tissues (*n* = 31) from Nanfang Hospital. The data are normalized to GAPDH. **E** Western blot was conducted to examine the protein levels of Myo1b in 8 cases of CRC tumor (T) and matched adjacent mucosa (N) tissues. The scatter diagram (right) shows the relative expression of Myo1b in human CRC tumors and matched adjacent mucosa tissues (normalized to β-actin). **F** Representative immunohistochemical staining of Myo1b protein in normal colonic mucosae, colon adenocarcinoma and paired adjacent mucosa with different staining intensities. **G, H** Survival curves for CRC patients with low Myo1b expression versus high Myo1b expression through analyzing data from GEO datasets (GSE14333 and GSE72970), and the optimal cut-off value of Myo1b expression was determined by Youden’s index. Each bar represented the mean ± SD (*n* ≥ 3). *p < 0.05, ***p < 0.001.

To identify the expression profiles of myosins in CRC, we firstly analyzed the expression levels of myosins using the data from GEO dataset (GSE106582). A Venn diagram was constructed, illustrating the overlap of differential expressed genes (DEGs) between 68 pairs CRC with adjacent mucosa tissues in GEO datasets (GSE106582) and myosin family genes (Fig. 1A) [17]. Figure 1B showed the heatmap representation of expression of myosins genes that were differently expressed between adjacent mucosa tissues and CRC tissues. And the Myo1b expression level was obviously increased in CRC tissues (Fig. 1C). Consistently, elevated Myo1b expression was confirmed in clinical specimens from NanFang Hospital both in mRNA and protein levels (Fig. 1D, E). Immunohistochemical (IHC) analysis showed that the expression of Myo1b was obviously higher in CRC tissues than that in normal tissues (Fig. 1F, Supplementary Table S3). In addition, we assessed the correlation between the expression of Myo1b with KRAS/BRAF mutation status and TNM stage in clinical CRC tissues using GEO database (GSE39084). As shown in Supplementary Table S4, the expression of Myo1b is closely associated with TNM stage and lymph node metastasis (N stage) in CRC patients (*P* < 0.05). However, it was not associated with the status of KRAS/BRAF mutation. Kaplan-Meier analysis was also used to determine the relationship between Myo1b expression and patient survival. The results indicated that higher levels of Myo1b expression were significantly correlated with the worse progression-free survival (PFS) and overall survival (OS) in other two GEO datasets (GSE14333 and GSE72970) (Fig. 1G, H).

**Fig. 2:**
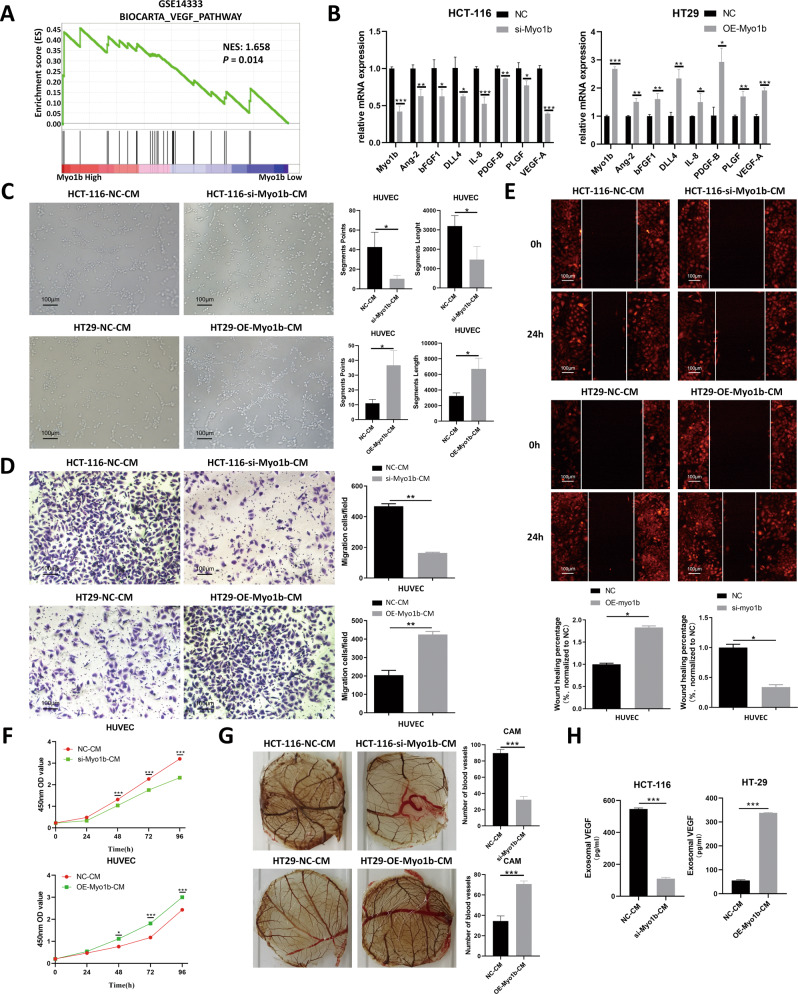
Myo1b promotes CRC angiogenesis by enhancing VEGF secretion of CRC cells. **A** Gene set enrichment analysis (GSEA) indicated that VEGF pathway was positively enriched in patients harboring high Myo1b expression from GEO datasets (GSE14333). **B** RT-qPCR confirmed the efficiency of si-Myo1b as well as OE-Myo1b in CRC cells and detected the effects of Myo1b on the expression of multiple angiogenesis-related factors in HCT-116 and HT29 cells. The conditioned medium (CM) of CRC cells (HCT-116 and HT29) with different Myo1b expression levels affected cell tube formation (**C**), migration (**D** & **E**) and proliferation (**F**) of HUVECs. **G** Blood vessels formed in representative images of the CAM assay after CM treatment. **H** The concentration of VEGF in the conditioned medium (CM) of HCT-116 and HT29 cells with different Myo1b expression levels was detected by ELISA assays. Each bar represented the mean ± SD (*n* ≥ 3). ^*^p < 0.05, ^**^p < 0.01, ^***^p < 0.001.

**Fig. 3:**
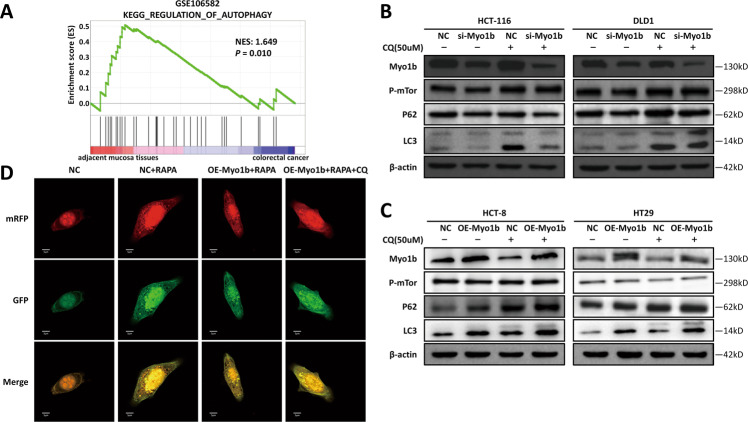
Myo1b mediates autophagy process by blocking autophagosome-lysosome fusion. **A** GSEA based on GEO dataset (GSE106582) indicated autophagy-related pathway was enriched in adjacent mucosa tissues when compared with colorectal cancer. **B, C** Western blot analyses of Myo1b, p-mTOR, P62 and LC3 in the CRC cells silencing or overexpressing Myo1b were performed in the presence or absence of Chloroquine (50 μM, 6 h). **D** Intensity of MAP1LC3 punctate in HCT-8 cells was analyzed after transfected with Myo1b over-expression vector or cultured with RAPA (500 nM, 24 h) and CQ (50 μM, 6 h).

Furthermore, we detected the expression of Myo1b in CRC cell lines using RT-qPCR and western blot, and the results revealed that majority of CRC cell lines exhibited the higher Myo1b expression at both the mRNA and protein levels compared with NCM460 normal epithelial cell line (Fig. S1). Based on the expression profiles in these CRC cells, low or high endogenous Myo1b expression cell lines were chosen to overexpress or silence Myo1b, respectively. Transfection efficiency of overexpression or knockdown in CRC cells was confirmed by RT-qPCR and western blot assays (Fig. 2B, and Fig. 3B, C). Subsequently, we explored the effect of Myo1b on biological behaviors of CRC cells. CCK-8 and colony formation assays were conducted to access the effect of Myo1b on cell proliferation in CRC cells. As indicated in Fig. S2a and S2b, Myo1b silencing markedly inhibited cell proliferation in HCT-116 and DLD1 cells. In contrast, Myo1b overexpression significantly enhanced the proliferative ability of HT29 and HCT-8 cells (Fig. S3a and S3b). In addition, transwell and wound healing assays were performed to examine the effect of Myo1b on the migration and invasion ability of CRC cells. As expected, the similar results that Myo1b expression was positively associated with the invasion and migration ability of CRC cells were also confirmed (Fig. S2c-S2f, and Fig. S3c-S3f). Collectively, these results suggest that the expression of Myo1b is enhanced in colorectal cancer, and it might play a crucial role in tumor progression.

### Myo1b promotes CRC angiogenesis by enhancing VEGF secretion of CRC cells

Although Myo1b has been proven to enhance cell migration and invasion in some previous studies, little is known about the effect of Myo1b on CRC angiogenesis. Interestingly, we performed the gene set enrichment analysis (GSEA) using the data from GEO dataset (GSE14333) to investigate the potential regulatory role of Myo1b in CRC and found that VEGF pathway was positively enriched in patients harboring high Myo1b expression (Fig. 2A). Therefore, the effect of Myo1b on the expression of multiple angiogenesis-related factors was detected in HCT-116 and HT29 cells by RT-qPCR. As shown in Fig. 2B, Myo1b knockdown obviously suppressed the mRNA expression of Ang-II, BFGF-B, DLL4, IL-8, PLGF, PDGF and VEGF-A in HCT-116 cell while Myo1b over-expression showed opposite phenomenon in HT29 cells, indicating that the potential of Myo1b in CRC angiogenesis.

For determining the effects of Myo1b on the formation of endothelial dependent vessels, an important composition in tumor vascularization, we conducted serials of assays to explore the effect of conditioned medium (CM) of CRC cells with different Myo1b expression levels on the biological behavior of HUVECs. As shown in Fig. 2C, HT29-Myo1b-overexpressed-CM promoted the tube formation of HUVECs while HCT-116-Myo1b-silenced-CM weaken the tube formation, compared with the NC-CM group respectively. Also, transwell and wound healing assays revealed that HT29-Myo1b-overexpressed-CM obviously reinforced the migration capacity of HUVECs, and this effect was opposite when Myo1b was silenced in HCT-116 cells (Figs. 2D and 2E). Consistently, CCK-8 assay showed that the CM from CRC cells with higher Myo1b expression level facilitated cell proliferation ability of HUVECs (Fig. 2F). In addition, the similar effect of Myo1b on angiogenesis was seen in CAM assay (Fig. 2G).

VEGF is a key secretory protein in regulating tumor vascularization. To verify whether Myo1b participated in CRC angiogenesis by mediating VEGF secretion of CRC cells, we detected the VEGF levels in the conditioned medium (CM) of CRC cells with different Myo1b expression levels by ELISA assays. The results showed that Myo1b silencing significantly reduced the secretion of VEGF of HCT-116 cells while over-expression of Myo1b in HT29 cells showed a reverse result. (Fig. 2H). In addition, a positive correlation between VEGF and Myo1b expression was found in CRC clinical tissue samples from GEO database (GSE14333, GSE72970) (Fig. S4). These results confirmed the important role of Myo1b on CRC angiogenesis which might be associated with enhancing VEGF secretion of CRC cells.

### Myo1b mediates autophagy process by blocking autophagosome-lysosome fusion

Autophagy is a major mechanism strongly associated with tumorigenesis in different types of cancer, including CRC [18]. As shown in Fig. 3A, GSEA based on the GEO dataset (GSE106582) was performed and the results showed that autophagy‐related genes were highly enriched in adjacent mucosa tissues, suggesting that autophagy plays an essential role in CRC. However, little is known about the role of Myo1b in the process of autophagy.

To clarify this issue, we assessed the influence of Myo1b on markers of autophagy and mTOR signaling pathway. Our results showed the decreased level of LC3-II and P62 in Myo1b-silenced CRC cells while the accumulation of LC3-II and P62 in Myo1b-overexpressed CRC cells (Fig. 3B, C). Interestingly, there was no obvious change of p-mTOR and total mTOR (unphosphorylated protein) expression in CRC cells overexpressing or silencing Myo1b (Fig. 3B, C, Fig. S5a, b), implying that Myo1b might participate in autophagy by affecting the autophagosome-lysosome fusion not the beginning of autophagy in CRC cells.

In order to investigate whether Myo1b blocked the fusion of autophagosomes and lysosomes, autophagic flux was monitored by tandem fluorescent tagged MAP1LC3 based on different pH stability conditions of GFP and RFP fluorescent proteins. As shown in Fig. 3d, in NC cells treated by RAPA (rapamycin), the intensity of RFP was stronger than that of GFP, and most of MAP1LC3 punctate were nearly red; while in Myo1b overexpressed cells treated by RAPA, the intensity of GFP was similar to RFP and approximately 100% of MAP1LC3 punctate were yellow (merging of green and red signals), suggesting an impaired autophagic flux. Taken together, these results suggested that Myo1b might participate in CRC cells autophagy progression by blocking autophagosome-lysosome fusion.

### Myo1b inhibits autophagic degradation of HIF-1α in CRC

**Fig. 4:**
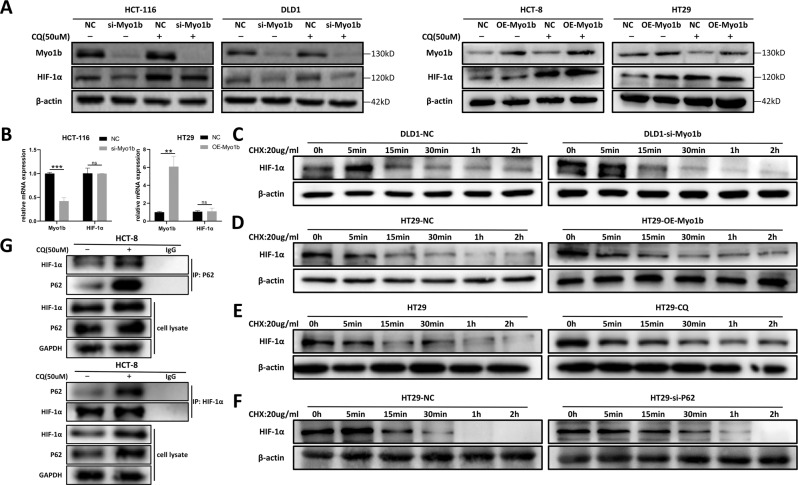
Myo1b inhibited autophagic degradation of HIF-1α in CRC. **A** Western blot was conducted to detect the effects of Myo1b expression and 50 μM CQ treatment for 6 h on HIF-1α protein expression in CRC cells. **B** RT-qPCR detects the effects of Myo1b on HIF-1α mRNA expression in HCT-116 and HT29 cells. Immunoblot assessment of HIF-1α stability in HCT-116 cells transfected with si-Myo1b **(C)** and HT29 cells transfected with over-expression vector **(D)**, si-P62 **(E)** or treated by 50 μM CQ for 6 h **(F)**. **G** Immunoprecipitation assay and western blot conducted to confirm the interaction between P62 and HIF-1α. Each bar represented the mean ± SD (*n* ≥ 3). ns, *p* < 0.05, ^**^p < 0.01, ^***^p < 0.001.

Tumor angiogenesis is an important process for tumor growth and metastasis and is usually related to hypoxia, and HIF-1α is a master gene in mediating different hypoxia associated cell processes including tumor angiogenesis [19, 20]. To verify whether HIF-1α participated in Myo1b-mediated angiogenesis in CRC, we performed western blot and RT-qPCR to detect the expression levels of HIF-1α protein and mRNA under different Myo1b expression levels. Importantly, Myo1b up-regulation increased while Myo1b down-regulation decreased the expression levels of HIF-1α protein (Fig. 4A) but not HIF-1α mRNA (Fig. 4B), indicating that Myo1b might modulate the expression of HIF-1α at the translational levels but not at the transcription levels. To further validate whether the synthesis or degradation of HIF-1α protein would be affected by Myo1b, we treated Myo1b knockdown or overexpression cells and control cells with protein synthesis inhibitor cycloheximide (CHX) and determined the expression levels of HIF-1α at varying time points. As expected, the half-life of HIF-1α in CHX-treated Myo1b silenced DLD1 cells was significantly shorter than that in control cells (Fig. 4C). Accordingly, Myo1b overexpressed HT29 cells showed a sluggish degradation of HIF-1α compared with control cells (Fig. 4D), suggesting that Myo1b might participate in the degradation of HIF-1α.

Protein homeostasis plays a fundamental role in cellular physiology and is strictly regulated by two different types of catabolic pathways, including the ubiquitin-proteasome system and the autophagy-lysosome system. HIF-1α has been reported to be selectively degraded by autophagy [21–24]. Based on this, we hypothesized that Myo1b might increase the protein expression of HIF-1α by inhibiting its autophagic degradation. Therefore, cycloheximide-based protein stability analysis was used to assess the effect of CQ (Chloroquine), the inhibitor of autophagy, on the degradation of HIF-1α. Consistently, CQ treatment obviously prolonged HIF-1α half-life compared to the mock group in HT29 cells (Fig. 4E). Furthermore, P62, which binds with the ubiquitinated proteins and tags them for autophagic degradation, was silenced in HT29 cells by siRNA to determine the autophagic degradation of HIF-1α and the results were in accordance with CQ treatment (Fig. 4F). Subsequently, coimmunoprecipitation (co-IP) assay was conducted to explore the protein interaction between HIF-1α and P62 in HCT-8 cells. The results showed that HIF-1α was interacted with P62 and the aggregate was accumulated under CQ treatment (Fig. 4G). Collectively, these results indicated that Myo1b inhibited autophagic degradation of HIF-1α in CRC cells.

### HIF-1α participates in Myo1b-mediated angiogenesis in CRC

**Fig. 5:**
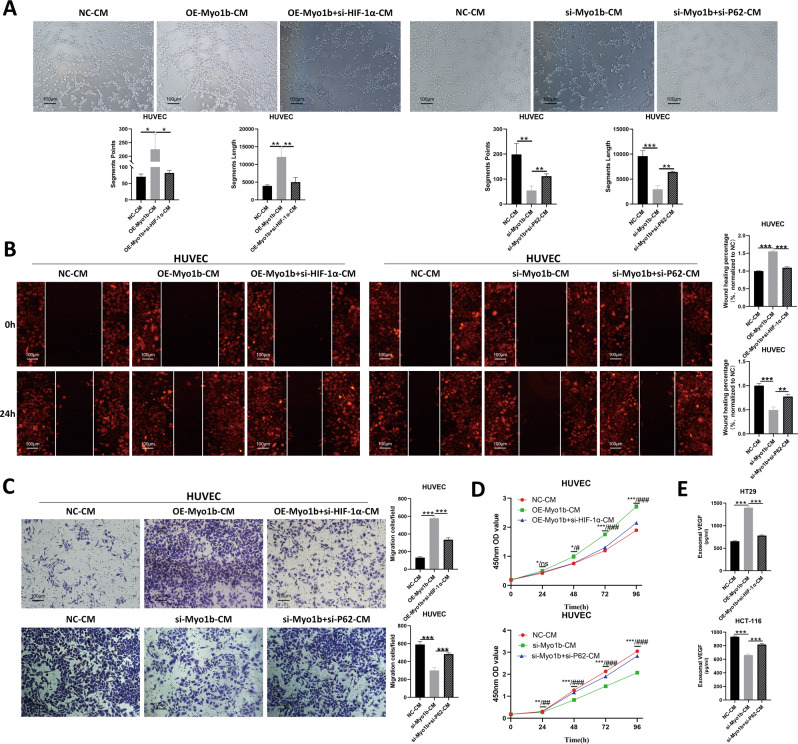
HIF-1α participated in the angiogenesis function of Myo1b in CRC. **A** Effects of si-P62 and si-HIF-1α on Myo1b mediated angiogenesis was analyzed through HUVEC tube formation assay. Wound healing assay **(B)** and transwell migration assay **(C)** were performed to explore the effects of si-P62 and si-HIF-1α on Myo1b mediated HUVEC migration. **D** Effects of CM treatment collected from indicated cells on HUVEC proliferation was evaluated by Cell Counting Kit-8 assays. **E** Effects of si-P62 and si-HIF-1α on Myo1b mediated VEGF secretion of HCT-116 and HT29 cells were measured using ELISA assay. Each bar represented the mean ± SD (*n* ≥ 3). *, **, ***: NC-CM VS OE-Myo1b-CM; #, ##, ###: OE-Myo1b-CM VS OE-Myo1b+si-HIF-1α-CM. *, #, *p* < 0.05; **, ##, *p* < 0.01; ***, ###, *p* < 0.001.

To determine whether Myo1b promoted CRC angiogenesis by inhibiting autophagic degradation of HIF-1α, we performed rescue experiments using siRNAs targeting HIF-1α and P62 and the transfection efficiency was confirmed by western blot (Fig. S6). As expected, HT29-Myo1b-overexpressed-CM obviously enhanced the tube formation ability of HUVECs which was reversed by si-HIF-1α, suggesting that the promoting angiogenesis function of Myo1b is attributable in most part to enhance HIF-1α activity (Fig. 5A). As demonstrated above, Myo1b inhibited autophagic degradation of HIF-1α in CRC cells. What’s more, the results showed that si-Myo1b CM inhibited tube formation, while co-depletion of P62 (si-P62 + si-Myo1b) largely abolished this effect (Fig. 5A). Furthermore, the results of wound healing assay and transwell assay indicated that HIF-1α knockdown markedly hindered the enhancement effect of HT29-Myo1b-overexpressed-CM on the migration ability of HUVECs, while si-P62 reversed the restraint of HCT-116-Myo1b-silenced-CM on HUVECs migration (Fig. 5B, C). Additionally, in CCK-8 assay, the effect of si-HIF-1α and si-P62 on Myo1b-mediated angiogenesis was present (Fig. 5D). More importantly, silencing HIF-1α dramatically inhibited the stimulatory effects of Myo1b on VEGF secretion (Fig. 5E). On the contrary, si-P62 obviously promoted VEGF secretion which was suppressed upon Myo1b knockdown (Fig. 5E). These findings underline the importance of HIF-1α in Myo1b-mediated angiogenesis in CRC cells.

### Myo1b promotes CRC xenograft tumors angiogenesis in vivo

**Fig. 6:**
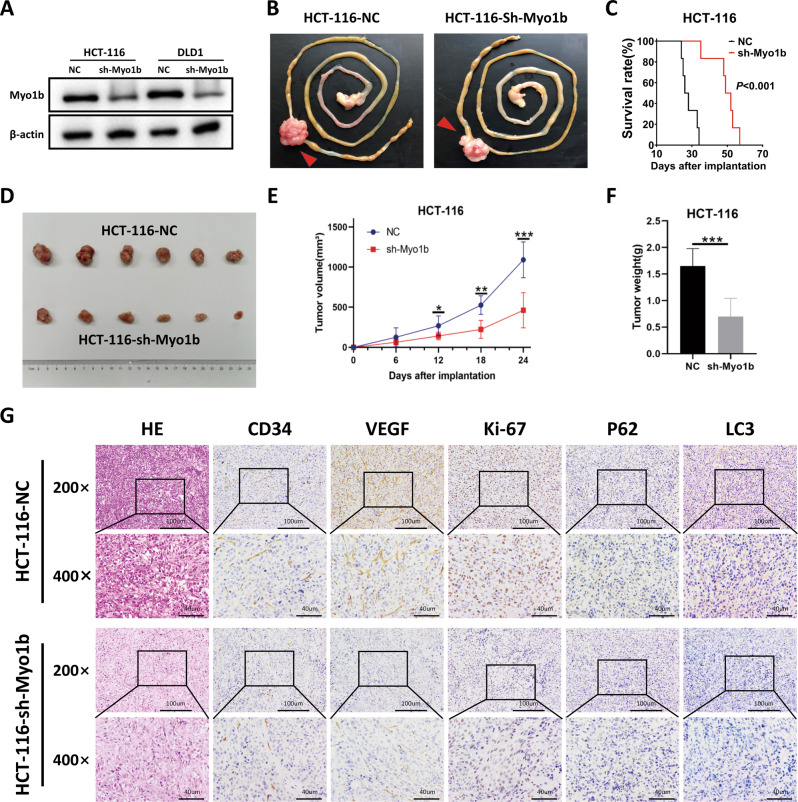
Myo1b promoted CRC xenograft tumors growth and angiogenesis in vivo. **A** Western blot was conducted to confirm the efficiency of sh-Myo1b in HCT-116 and DLD1 cells. **B** Images of CRC orthotopic tumors from BALB/c nude mice after the orthotopic injection of stable Myo1b-knockdown HCT-116 cells (sh-Myo1b) or control HCT-116 cells (NC). **C** Kaplan–Meier survival analysis of mice bearing orthotopic xenografts. *n* = 6 for each group. **D** Images of xenograft tumors from groups of BALB/c nude mice 24 days after the subcutaneous injection of stable Myo1b-knockdown HCT-116 cells (sh-Myo1b) or control HCT-116 cells (NC). **E** Subcutaneous tumor size was monitored every 6 days after injection. **F** Weights of subcutaneous xenograft tumors were measured after sacrificed at day 24. **G** Representative images of HE and IHC staining with antibody targeting human CD34, VEGF, Ki67, P62 and LC3 in subcutaneous xenograft tumors. Original magnification, ×200 or ×400; Scale bar, 100 μm or 40 μm. Each bar represented the mean ± SD (*n* ≥ 3). ^*^p < 0.05, ^**^p < 0.01, ^***^p < 0.001.

To determine if Myo1b could affect tumor growth and angiogenesis in vivo, particularly during tumorigenesis, HCT-116 cells stably transfected by NC or sh-Myo1b vector were injected orthotopically and subcutaneously in nude mice to establish the xenograft models and the transfection efficiency was confirmed by western blot (Fig. 6A). The survival time of orthotopic xenograft tumor mice was analyzed with a Kaplan–Meier curve and the results revealed that Myo1b knockdown significantly prolonged survival time of tumor-bearing nude mice (Fig. 6B, C). The growth of subcutaneous tumor sizes was monitored every six days after inoculation for 24 days. All subcutaneous mice were sacrificed at 24th days after inoculation and the tumors were weighed. In accordance with the results in vitro, silencing Myo1b dramatically inhibited tumor growth compared with NC group (Fig. 6D–F). Immunohistochemical staining for CD34 was performed to detect the density of endothelial dependent vessels (EDVs) in subcutaneous xenograft tumors and the results showed that the EDVs density was significantly decreased in Myo1b silenced tumors. Notably, the VEGF secretion was obviously inhibited when Myo1b was silenced. Moreover, histological analysis of Myo1b-silenced xenografts showed markedly decreased levels of proliferation marker Ki-67 and autophagy marker LC3 and P62 (Fig. 6E). These results supported a positive regulatory role of Myo1b on tumor growth and angiogenesis in vivo.

## Discussion

Colorectal cancer is a common tumor characterized by its high mortality. However, the underlying molecular mechanisms that drive CRC tumorigenesis are largely unclear [25]. Myosins represent a large family of actin motor proteins that play key roles in regulating actin cytoskeleton architecture and dynamics [5]. Mounting evidence highlights several members of the myosin superfamily as important regulators of CRC. Recent studies show that upregulation of Myo6 in CRC samples and its high expression is associated with prognostic of poor survival [26, 27]. Myh9 promotes cancer cell growth and metastasis via activation of MAPK/AKT signaling in CRC [28]. In this study, we explored the potential function of Myo1b in CRC and confirmed that Myo1b facilitated CRC progression and angiogenesis by inhibiting autophagic degradation of HIF-1α, suggesting that Myo1b acts as a tumor promoter in CRC.

Myo1b is one member of the unconventional class I myosins family including eight different motors (Myo1a through Myo1h). Due to a dual affinity to actin and membrane phospholipids, class I myosins can tether actin filaments to the cellular membranes and exhibit the broad functions in regulating membrane tension, cell–cell and cell–matrix adhesions and intracellular vesicle trafficking [17, 29]. It has been verified that two members of the myosin I family (Myo1a and Myo1d) are implicated with CRC development. Low Myo1a expression in CRC is shown to be a prognostic marker of poor patient survival which may be associated with frequent frameshift mutations or promoter hypermethylation [30]. And Myo1d contributes to colorectal carcinogenesis possibly as a novel oncogene and thus may serve as an additional target for suppression of RTK signaling in cancer treatment [31]. Through the integrative analysis of GEO data (GSE106582, GSE14333, GSE72970, GSE39084), we observed that Myo1b was obviously overexpressed in CRC tissues and its high expression was significantly related to poor prognosis of CRC patient, which was further validated in 8 matched human CRC and normal tissue specimens using western blot, RT-qPCR and IHC. Subsequently, in vitro and in vivo data support that Myo1b promoted cell proliferation, migration and invasion in CRC cells. Interestingly, Rai et al. demonstrated that fibroblasts activated by late-stage cancer-exosomes (SW620-Exos) display elevated expression of proteins (PDLIM1, MYO1B, MMP11, EMMPRIN, ADAM10) that support invasive outgrowth and metastasis, which is consistent with our conclusions on the importance of Myo1b in facilitating CRC progression [32]. These findings emphasize the potential clinical significance of Myo1b, and indicate that further investigations are needed to evaluate its diagnostic value for CRC patients.

Although the dysregulation of Myo1b expression has been extensively investigated in the pathological processes of different tumors [6, 8, 9, 11, 13–15], the functional mechanism of Myo1b in CRC progression has rarely been explored. Our study provided evidence that supports the roles of Myo1b in CRC cell proliferation, migration and invasion. More importantly, we demonstrated for the first time that Myo1b promoted tumor angiogenesis. Although angiogenesis is essential for tumor growth and metastasis, whether the effect of Myo1b on the invasion/motility of CRC cells is associated with its proangiogenic roles is an interesting topic that needs to be further investigated. In the present study, we mainly focused on the effect of Myo1b on CRC angiogenesis and its underlying mechanism. It is still unclear whether the signaling pathways involved in angiogenesis are regulated by Myo1b despite that VEGF pathway was positively enriched in patients harboring high Myo1b expression by GSEA analysis. Our findings showed that many of angiogenesis-related factors are downstream targets of Myo1b. Later, in vitro and in vivo experiments confirmed that Myo1b plays an important role in CRC angiogenesis and regulates VEGF expression. We further collected supernatants from the Myo1b-silenced HCT-116 cells and Myo1b-overexpressed HT29 cells to use as CM (conditioned medium) to stimulate HUVECs. Accordingly, CM from si-Myo1b CRC cells significantly inhibited the proliferation, migration and tube formation of HUVECs in vitro. In contrast, Myo1b overexpression in CRC cells had the opposite impact on HUVECs. Furthermore, in vivo CAM experiments with induced neovascularization also support our assumption in vitro findings. Meanwhile, VEGF secretion was markedly inhibited when Myo1b was silenced in HCT-116 cells according to the ELISA results. These data indicate that Myo1b can potentiate VEGF secretion and promote angiogenesis in CRC.

It has been well documented dysregulation of autophagy is an important event for initiation and development of CRC [33]. Only a few myosins have so far been reported to function as critical regulators of autophagy. Non-muscle myosin IIA functions in the early stages delivering membrane for the initial formation of the autophagosome, whereas Myo1c and Myo6 are involved in the final stages providing specific membranes for autophagosome maturation and its fusion with the lysosome [34]. Recent research demonstrated that Myo1b mediates the effect of Arg-II in activating mTORC1-S6K1 through promoting peripheral lysosomal positioning, which results in spatial separation and thus dissociation of TSC from lysosome [35]. Here we revealed that either overexpressing or silencing Myo1b had no effect on the mTOR signaling pathway in CRC cells. Nevertheless, our data indicated that the expression of LC3 and P62 exhibited the same tendency following the change of Myo1b, which revealed that Myo1b might participate in the autophagy of CRC cells by blocking autophagosome-lysosome fusion. Additionally, supporting this notion, Myo1b overexpression was able to increase the accumulation of yellow MAP1LC3 puncta (merging of green and red signals).

HIF-1α is one of the response elements to help cells to adapt to hypoxia, which regulates vascularization and angiogenesis as well as various metabolic pathways [21]. It was mainly degraded by ubiquitin–proteasome system [36]. The HIF-1α/VEGF axis has been emphasized as a crucial component of angiogenesis in multiple malignancies [20, 37, 38]. According to the aforementioned findings that elevated expression of Myo1b promotes angiogenesis and enhances VEGF secretion of CRC cells, further studies are required to determine the molecular mechanism. In the present study, we found that Myo1b could modulate the expression of HIF-1α at the translational levels but not at the transcription levels. Also, HIF-1α might be regulated by Myo1b via inhibiting autophagic degradation of HIF-1α in CRC cells. In fact, the autophagy pathway has also been implicated for oxygen concentration independent HIF-1α degradation. For example, Nkx3.2 facilitates HIF-1α degradation via a macroautophagy (MA) pathway mediated by P62/SQSTM1[21]. Considering the roles of Myo1b in regulating autophagy and lysosomal positioning, we then tried to determine whether HIF-1α degradation is associated with the Myo1b-mediated autophagy. Interestingly, our results showed that P62 binds to HIF-1α, causing HIF-1α degradation through the lysosomal pathway, which is consistent with the previously published studies [21, 24]. Besides, si-P62 succeeded to rescue the inhibiting effect of Myo1b knockdown on angiogenesis, while si-HIF-1α markedly blocked the augment effect of Myo1b overexpression on angiogenesis. Hence, these results highlight the importance of HIF-1α/VEGF pathway in Myo1b-mediated angiogenesis in CRC cells, and reveal a novel degradation pathway of HIF-1α by Myo1b-mediated macroautophagic lysosomal pathway.

## Conclusions

**Fig. 7:**
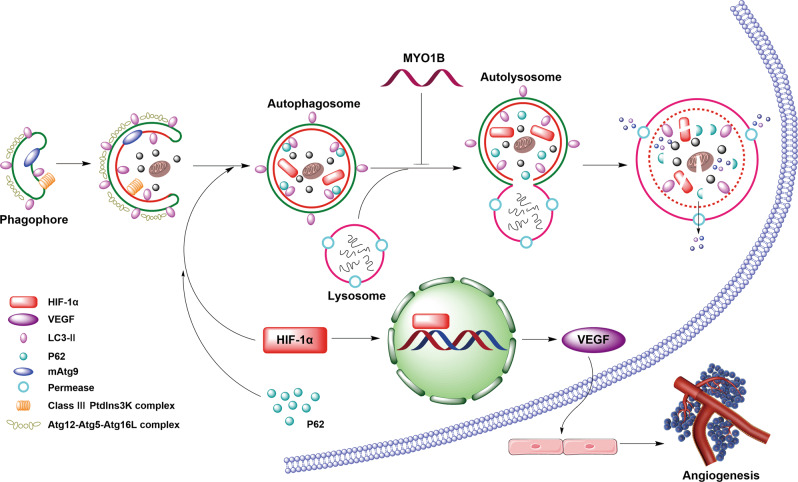
Schematic diagram of the mechanism on Myo1b mediated autophagy and angiogenesis in CRC. The high-expression of Myo1b in CRC cells disrupts the fusion between autophagosomes and lysosomes, which blocks the selective degradation of HIF-1α. The accumulated HIF-1α further upregulates the proangiogenic related genes and consequently promotes angiogenesis in CRC.

In summary, we show that Myo1b is upregulated in CRC tissues and the enhanced expression of Myo1b is associated with poor prognosis. Functional studies indicate that Myo1b promotes CRC angiogenesis and metastasis. Mechanistically, Myo1b functions as a positive regulator of HIF-1α/VEGF pathway by disrupting the fusion between autophagosomes and lysosomes, blocking the selective degradation of HIF-1α and then leading to the accumulation of HIF-1α (Fig. 7). Therefore, our study suggests that Myo1b may severe as a feasible target for CRC management, and reveal novel insights into the role of Myo1b in CRC progression.

## Materials and methods

### Cell culture and human tissue samples

Human umbilical vein endothelial cells (HUVEC), human colorectal cancer cell lines HCT-116, HCT-8, HT29, DLD1, HCT-15, RKO, SW480, SW620 and human normal colon mucosal epithelial cell line NCM460 were purchased from the CBCC (Shanghai, China). All cells were cultured in RPMI 1640 (Gibco, Carlsbad, CA, USA) with 10% fetal bovine serum (FBS, Gibco, Carlsbad, CA, USA), 100 IU/mL penicillin G and 100 μg/mL streptomycin (Invitrogen Life Technologies, Carlsbad, CA, USA). Cells were maintained in a humidified atmosphere containing 5% CO_2_ at 37 ˚C. All cell lines used in this study obtained certificates within four years that authenticated by performing short tandem repeat (STR) profiling, and experiments were performed in cells propagated less than 6 months after cell resuscitation.

A tissue microarray comprising of 4 normal colonic mucosae, 28 colon adenocarcinoma and paired adjacent mucosa was purchased from Biomax (US Rockville, MD). 8 matched human CRC and normal tissue specimens for western blot analysis, while 31 pairs tissue specimens for qRT-PCR analysis were obtained from patients undergoing surgical treatment in Nanfang Hospital, Southern Medical University between 2018 and 2020. This study was approved by the Ethical Committee of Southern Medical University, and all aspects of the study complied with the Declaration of Helsinki. The expression of target genes profiling studies on CRC samples were identified through searching in GEO: GSE106582, GSE14333, GSE72970, GSE39084.

### RNA isolation, reverse transcription, and quantitative real-time PCR

Total RNA in cells or tissues was extracted using RNAiso Plus reagent (Takara). To quantify the expression of Myo1b and proangiogenic factors, total RNA was subjected to polyadenylation and reverse transcription (RT) using a PrimeScript RT reagent kit (RR047A, TaKaRa). Real-time polymerase chain reaction (PCR) analysis was carried out using a TB Green^TM^ Premix Ex Taq^TM^ (Tli RNaseH Plus; RR420A; Takara) on an ABI 7500HT system according to the manufacturer’s instructions. GAPDH was used as an endogenous control. All samples were normalized to internal controls, and fold changes were calculated through relative quantification (2^-ΔΔCt^). The primers used are listed in Supplementary Table S1.

### Western blot and co-immunoprecipitation

Radio-immunoprecipitation assay lysis buffer supplemented with protease and phosphorylase inhibitors cocktail was used to extract total proteins from cells or tissues. Proteins were quantified by BCA protein assay kit (Pierce, KeyGEN BioTECH, Jiangsu, China), separated by SDS-PAGE gel and then transferred onto the PVDF membrane. Tris buffer containing 0.1% Tween-20 and 5% nonfat milk was used to block the membrane at 4 °C for 2 h. The primary antibodies specific to Myo1b (1:1000 dilution; Abcam, Inc, Cambridge, MA, USA), P62, LC3, p-mTOR, mTOR, GAPDH, β-actin (1:3000 dilution; Proteintech Group, Inc, Chicago, IL, USA) and HIF-1α (1:1000 dilution, CST, Inc, MA, USA) were used to incubate with the membrane overnight, followed by the treatment of HRP-conjugated secondary antibody (anti-rabbit IgG/anti-mouse IgG, 1:3000 dilution; Proteintech Group, Inc, Chicago, IL, USA) at room temperature (RT) for 2 h. The signal was detected by the enhanced chemiluminescence detection system (Tennon5200, shanghai, China) following the manufacturer.

In co-immunoprecipitation assay, cell extracts were incubated 2 h at 4 °C with IgG and protein A/G-agarose to get rid of non-specific binding. The rabbit IgG and primary antibodies against HIF-1α (CST, Inc, MA, USA), and P62 (Proteintech Group, Inc, Chicago, IL, USA) were then added to separate cell extract tubes with protein A/G-agarose for incubation at 4 °C overnight. The protein A/G-agarose was collected by centrifugation. Immuno-precipitated proteins were analyzed by western bolt.

All the western bolt showed are representative of at least three independent experiments.

### Immunohistochemistry (IHC)

Specimens of surgical tumor tissues from CRC patients and xenograft tumor tissues were fixed with 4% formalin, paraffin embedded and sectioned (4 μm). Then the tissue sections were deparaffinized and dehydrated followed by incubation in 3% hydrogen peroxide for 10 min. Slides were stained with primary antibodies against Myo1b (Abcam, 1:200), Ki-67 (1:300, servicebio), CD34 (1:200, servicebio), P62 (1:200, servicebio), VEGF (1:200, servicebio), LC3 (1:200, proteintech) at 4 °C overnight after blocking with 5% BSA in PBS for 1 h at room temperature. Corresponding secondary antibodies were used for 1 h at RT. Targeted molecules were detected following DAB staining for immunohistochemistry. Slides were finally counterstained with hematoxylin. The staining intensity of immunostained sections was evaluated by two independent pathologists who were blinded to the patients’ clinical information.

### Cell transfection

The small interfering RNAs (siRNAs) separately targeting Myo1b (si-Myo1b), HIF-1α (si‐HIF-1α), P62 (si‐P62), and the scrambled oligonucleotide control (siRNA-NC) were purchased from GenePharma (Shanghai, China). The short hairpin RNA (shRNA) vector specific to Myo1b (sh‐Myo1b) and a scrambled negative control vector were obtained from Genechem (Shanghai, China). Detailed information on all siRNAs sequences was provided in Supplementary Table S2. To construct an overexpression plasmid, the full‐length of CDS of Myo1b (NCBI: NM_001161819) was cloned into the pcDNA3.1 vector by GeneCopoeia (Guangzhou, China). Cell transfection was performed using Lipofectamine^TM^ 2000 (Invitrogen) according to the instruction.

### Cell Counting Kit‐8 (CCK‐8) assay and colony formation

For CCK‐8 assay, a total of 3,000 cells were seeded in a 96‐well plate in a volume of 100 μl complete medium containing 10% FBS per well each with five replicate wells. At 0, 24, 48, 72, and 96 h after transfection, 90 μl 1640 and 10 μl CCK‐8 (Dojindo Laboratory, Japan) were added to each well and incubated for another 1.5 h. The absorbance at 450 nm was detected with a microplate reader (Biotex, USA).

For colony formation, cells (2 × 10^3^ cells/well) were cultured in a six‐well plate with complete medium. After 14 days, the colonies were fixed in methanol and then stained with 0.1% crystal violet (Keygen, Nanjing, China).

### Wound healing assay

Cells were seeded in six‐well plate and cultured for 24 h after transfection. A sterile 10 µl pipette tip was used to scratch the monolayers making artificial homogeneous wounds. After washing with PBS, the wounded cells were cultured in serum‐free medium. Images were captured at 0 and 24 h using an inverted phase contrast microscope.

### Cell migration and invasion assay

Migration and invasion assays were performed using cell culture insert with 8 um pores in 24-well plates (Costar, USA). The upper chamber was coated with (for invasion assay) or without (for migration assay) 50 μl Matrigel (BD Biosciences, San Jose, CA, USA). A total of 5 × 10^4^ Cells were trypsinized and resuspended in 200 μl serum-free medium and seeded onto the upper chamber, while the lower chamber was filled with 0.6 mL medium containing 20% FBS. After incubation at 37 °C for 24 or 48 h, migrated or invaded cells attached to the bottom surface of the insert were then fixed with methanol and stained with crystal violet. Penetrated cells were photographed under a light microscope in five random visual fields.

### Human umbilical vein endothelial cells (HUVEC) tube formation assay

After unfreezing overnight, 50 μl matrigel matrix (BD Biosciences, USA) was plated in 96-well plate and incubated at 37 °C for 30 min for polymerizing matrigel. HUVECs resuspended in 200 μl medium with 10% FBS were seeded on the matrigel-coated well. The plate was then incubated at 37 °C in 5% CO_2_ humidified atmosphere 4 h before image taking. The tube formation ability was determined by measuring segments points and segments length. Each experiment was repeated three times.

### Chicken chorioallantoic membrane (CAM) assay

Day-6 fertilized chicken eggs (Yueqin Breeding Co. Ltd, Guangdong, China) were chosen to perform the CAM assay. For exposing the CAM, a window about 1.0 cm in diameter was opened in the eggshell. A sterile rubber ring in 0.5 cm diameter was placed on the CAM through the window and then 100 μl conditioned medium (CM) was added into the ring. After closing the window using a piece of sterile adhesive tape, eggs were placed in a 37 °C incubator with 80–90% relative humidity for 2-3 days. CAMs were fixed by stationary solution (methanol: acetone=1:1) for 15 min before cut and harvest. The effect of CM on angiogenesis was evaluated through assessing the number of second- and third-order vessels.

### Enzyme-linked immunosorbent assay

To promote the secretion of VEGF, CRC cells were cultured with serum‐free medium for 24 h. Then the culture medium was collected and the cell number was counted. The human VEGF ELISA Kit (Enzyme-linked Biotechnology, Shanghai, China) was used to measure the total levels of extracellular VEGF in the supernatant according to the manufacturer’s introductions. The cytokine expression level (pg/ml) per 10^6^ cells was analyzed.

### Confocal microscope

HCT-8 cells were plated in 6-well plates and transfected with mRFP-GFP-LC3 adenoviral vectors (HanBio Technology, Shanghai, China) according to the manufacturer’s instructions when reaching 50%–70% confluence. The principle of the assay is based on different pH stability of green and red fluorescent proteins. When autophagosomes combine with lysosome, the fluorescent signal of GFP could be quenched because of the acidic condition (pH below 5), while the mRFP fluorescent signal has no significant change under the acidic condition. In green and red-merged images, because of the merge of RFP and GFP, autophagosomes are shown as yellow puncta, while autolysosomes are shown as red puncta for the quenching of GFP. Autophagic flux is enhanced when more of both yellow and red puncta are shown in cells, while autophagic flux is blocked when only yellow puncta are increased without red puncta increased, or when both yellow and red puncta are decreased in cells [39]. After transfected with the adenoviruses, CRC cells were seeded on confocal disks and transfected with Myo1b overexpression vector or control vector, respectively. After treating with rapamycin (RAPA) and Chloroquine (CQ), the LC3 puncta were examined with laser scanning confocal microscope (Nikon, Japan).

### In vivo tumor xenograft study

#### Subcutaneous xenograft colorectal cancer mouse model

HCT-116 cells stably transfected with sh-Myo1b or control vector were subcutaneously injected into Five-week-old BABL/c male nude mice, which were purchased from the Central Animal Facility of Southern Medical University. Each group included 6 mice. Tumor growth was monitored every 6 days after injection. All mice were sacrificed when having been monitored for 24 days and the final tumor weight was measured. Then the xenografts were fixed and sectioned for hematoxylin–eosin (HE) and immunohistochemical analysis. All the protocols in the study have been approved by the Animal Care and Use Committee of Southern Medical University.

#### Orthotopic xenograft colorectal cancer mouse model

HCT-116 cells stably transfected with sh-Myo1b or control vector were prepared and suspended by fresh PBS to a concentration 1 × 10^6^ cells/50 µL. Six-week-old BABL/c male nude mice were purchased from the Central Animal Facility of Southern Medical University. Each group included 6 mice. After anesthesia, laparotomy was performed on the mice to expose the cecum. A 50 µL volume of cells were slowly injected into the cecal wall. Removed the needle carefully and made sure that the injection point is not leaking. Gently returned the cecum back to the abdominal cavity and then sutured the abdominal wall and skin. Mice with moribund appearance were sacrificed. Orthotopic xenograft CRC masses were measured and harvested for further study. All the protocols in the study have been approved by the Animal Care and Use Committee of Southern Medical University.

### Statistical analysis

The data were analyzed using SPSS version 20.0 software (SPSS, Chicago, IL, USA). Sample size for each study was chosen on the basis of literature documentation of similar well-characterized experiments, and no statistical method was used to predetermine sample size. The clinical data were analyzed using nonparametric tests. Survival curves of Myo1b expression in CRC patients were analyzed using the Kaplan–Meier method and compared by the Log-rank test. Pearson’s chi-squared (χ^2^) test and unpaired Student’s *t*-test were used to evaluate the significance of the differences among different groups. All statistical tests were two-sided. All data were presented as means ± SD (standard deviation).

## Supplementary information

supplemental figure

supplemental table

Full and uncropped western blots

aj-checklist

## Data Availability

The datasets used and analyzed during the current study are available from the corresponding author on reasonable request.

## References

[1] Siegel RL, Miller KD, Fuchs HE, Jemal A (2021). Cancer Statistics, 2021. CA: cancer J clinicians.

[2] Montminy EM, Zhou M, Maniscalco L, Abualkhair W, Kim MK, Siegel RL (2021). Contributions of Adenocarcinoma and Carcinoid Tumors to Early-Onset Colorectal Cancer Incidence Rates in the United States. Ann Intern Med.

[3] Siegel RL, Miller KD, Goding Sauer A, Fedewa SA, Butterly LF, Anderson JC (2020). Colorectal cancer statistics, 2020. CA: a cancer J clinicians.

[4] Sun D, Zhang F, Qian J, Shen W, Fan H, Tan J (2018). 4′-hydroxywogonin inhibits colorectal cancer angiogenesis by disrupting PI3K/AKT signaling. Chem-Biol Interact.

[5] Naydenov N, Lechuga S, Huang E, Ivanov A (2021). Myosin Motors: Novel Regulators and Therapeutic Targets in Colorectal Cancer. Cancers.

[6] Makowska KA, Hughes RE, White KJ, Wells CM, Peckham M (2015). Specific Myosins Control Actin Organization, Cell Morphology, and Migration in Prostate Cancer Cells. Cell Rep.

[7] Arjonen A, Kaukonen R, Mattila E, Rouhi P, Högnäs G, Sihto H (2014). Mutant p53–associated myosin-X upregulation promotes breast cancer invasion and metastasis. J Clin Investig.

[8] Chapman BV, Wald AI, Akhtar P, Munko AC, Xu J, Gibson SP (2015). MicroRNA-363 targets myosin 1B to reduce cellular migration in head and neck cancer. BMC Cancer.

[9] Yamada Y, Koshizuka K, Hanazawa T, Kikkawa N, Okato A, Idichi T (2018). Passenger strand of miR-145-3p acts as a tumor-suppressor by targeting MYO1B in head and neck squamous cell carcinoma. Int J Oncol.

[10] Ohmura G, Tsujikawa T, Yaguchi T, Kawamura N, Mikami S, Sugiyama J (2015). Aberrant Myosin 1b Expression Promotes Cell Migration and Lymph Node Metastasis of HNSCC. Mol Cancer Res.

[11] Wen LJ, Hu XL, Li CY, Liu J, Li ZY, Li YZ (2021). Myosin 1b promotes migration, invasion and glycolysis in cervical cancer via ERK/HIF-1α pathway. Am J Transl Res.

[12] Zhang H-R, Lai S-Y, Huang L-J, Zhang Z-F, Liu J, Zheng S-R (2018). Myosin 1b promotes cell proliferation, migration, and invasion in cervical cancer. Gynecologic Oncol.

[13] Shimonosono M, Idichi T, Seki N, Yamada Y, Arai T, Arigami T (2019). Molecular pathogenesis of esophageal squamous cell carcinoma: Identification of the antitumor effects of miR‑145‑3p on gene regulation. Int J Oncol.

[14] Zhou X, Wang R, Li X, Yu L, Hua D, Sun C (2019). Splicing factor SRSF1 promotes gliomagenesis via oncogenic splice-switching of MYO1B. J Clin Investig.

[15] Xie L, Huang H, Zheng Z, Yang Q, Wang S, Chen Y (2021). MYO1B enhances colorectal cancer metastasis by promoting the F-actin rearrangement and focal adhesion assembly via RhoA/ROCK/FAK signaling. Ann Transl Med.

[16] Ciccone V, Terzuoli E, Donnini S, Giachetti A, Morbidelli L, Ziche M (2018). Stemness marker ALDH1A1 promotes tumor angiogenesis via retinoic acid/HIF-1α/VEGF signalling in MCF-7 breast cancer cells. J Exp Clin Cancer Res.

[17] Masters TA, Kendrick-Jones J, Buss F (2017). Myosins: Domain Organisation, Motor Properties, Physiological Roles and Cellular Functions. Handb Exp Pharm.

[18] Koustas E, Sarantis P, Kyriakopoulou G, Papavassiliou AG, Karamouzis MV (2019). The Interplay of Autophagy and Tumor Microenvironment in Colorectal Cancer—Ways of Enhancing Immunotherapy Action. Cancers.

[19] Chen L-Y, Wang L, Ren Y-X, Pang Z, Liu Y, Sun X-D (2020). The circular RNA circ-ERBIN promotes growth and metastasis of colorectal cancer by miR-125a-5p and miR-138-5p/4EBP-1 mediated cap-independent HIF-1α translation. Mol Cancer.

[20] Cao D, Hou M, Guan YS, Jiang M, Yang Y, Gou HF (2009). Expression of HIF-1alpha and VEGF in colorectal cancer: association with clinical outcomes and prognostic implications. BMC Cancer.

[21] Im S, Kim D-W (2017). Nkx3.2 induces oxygen concentration-independent and lysosome-dependent degradation of HIF-1α to modulate hypoxic responses in chondrocytes. Cell Signal.

[22] Fujita N, Chiba K, Shapiro IM, Risbud MVHIF-1α (2012). and HIF-2α degradation is differentially regulated in nucleus pulposus cells of the intervertebral disc. J Bone Miner Res.

[23] Hubbi ME, Hu H, Kshitiz, Ahmed I, Levchenko A, Semenza GL (2013). Chaperone-mediated autophagy targets hypoxia-inducible factor-1alpha (HIF-1alpha) for lysosomal degradation. J Biol Chem.

[24] Liu X-W, Cai T-Y, Zhu H, Cao J, Su Y, Hu Y-Z (2014). Q6, a novel hypoxia-targeted drug, regulates hypoxia-inducible factor signaling via an autophagy-dependent mechanism in hepatocellular carcinoma. Autophagy.

[25] Cao M, Wang Y, Xiao Y, Zheng D, Zhi C, Xia X (2021). Activation of the clock gene TIMELESS by H3k27 acetylation promotes colorectal cancer tumorigenesis by binding to Myosin-9. J Exp Clin Cancer Res.

[26] You W, Tan G, Sheng N, Gong J, Yan J, Chen D (2016). Downregulation of myosin VI reduced cell growth and increased apoptosis in human colorectal cancer. Acta Biochimica et Biophysica Sin.

[27] Luan Y, Li X, Luan Y, Zhao R, Li Y, Liu L (2020). Circulating lncRNA UCA1 Promotes Malignancy of Colorectal Cancer via the miR-143/MYO6 Axis. Mol Ther Nucl Acids.

[28] Wang B, Qi X, Liu J, Zhou R, Lin C, Shangguan J (2019). MYH9 Promotes Growth and Metastasis via Activation of MAPK/AKT Signaling in Colorectal Cancer. J Cancer.

[29] McIntosh BB, Ostap EM (2016). Myosin-I molecular motors at a glance. J Cell Sci.

[30] Mazzolini R, Dopeso H, Mateo-Lozano S, Chang W, Rodrigues P, Bazzocco S (2012). Brush border Myosin Ia has tumor suppressor activity in the intestine. Proc Natl Acad Sci.

[31] Ko Y-S, Bae JA, Kim KY, Kim SJ, Sun EG, Lee KH (2019). MYO1D binds with kinase domain of the EGFR family to anchor them to plasma membrane before their activation and contributes carcinogenesis. Oncogene.

[32] Rai A, Greening DW, Chen M, Xu R, Ji H, Simpson RJ (2019). Exosomes Derived from Human Primary and Metastatic Colorectal Cancer Cells Contribute to Functional Heterogeneity of Activated Fibroblasts by Reprogramming Their Proteome. PROTEOMICS.

[33] Devenport SN, Shah YM (2019). Functions and Implications of Autophagy in Colon Cancer. Cells.

[34] Kruppa AJ, Kendrick Jones J, Buss F (2016). Myosins, Actin and Autophagy. Traffic.

[35] Yu Y, Xiong Y, Montani J-P, Yang Z, Ming X-F (2018). Arginase-II activates mTORC1 through myosin-1b in vascular cell senescence and apoptosis. Cell Death Dis.

[36] Maxwell PH, Wiesener MS, Chang GW, Clifford SC, Vaux EC, Cockman ME (1999). The tumour suppressor protein VHL targets hypoxia-inducible factors for oxygen-dependent proteolysis. Nature.

[37] Palazon A, Tyrakis PA, Macias D, Veliça P, Rundqvist H, Fitzpatrick S (2017). An HIF-1α/VEGF-A Axis in Cytotoxic T Cells Regulates Tumor Progression. Cancer Cell.

[38] Xiang Z-L, Zeng Z-C, Fan J, Tang Z-Y, Zeng H-Y, Gao D-M (2011). Gene Expression Profiling of Fixed Tissues Identified Hypoxia-Inducible Factor-1α, VEGF, and Matrix Metalloproteinase-2 as Biomarkers of Lymph Node Metastasis in Hepatocellular Carcinoma. Clin Cancer Res.

[39] Zhou C, Zhong W, Zhou J, Sheng F, Fang Z, Wei Y (2012). Monitoring autophagic flux by an improved tandem fluorescent-tagged LC3 (mTagRFP-mWasabi-LC3) reveals that high-dose rapamycin impairs autophagic flux in cancer cells. Autophagy.

