# Supplementary material for: Myo1b promotes tumor progression and angiogenesis by inhibiting autophagic degradation of HIF-1α in colorectal cancer
Source: Cell Death Dis. 2022 Nov 8;13(11):939. doi: 10.1038/s41419-022-05397-1 (PMC9643372; doi:10.1038/s41419-022-05397-1)
Supplement: Supplementary file 1 — supplemental figure [file 41419_2022_5397_MOESM1_ESM.docx]

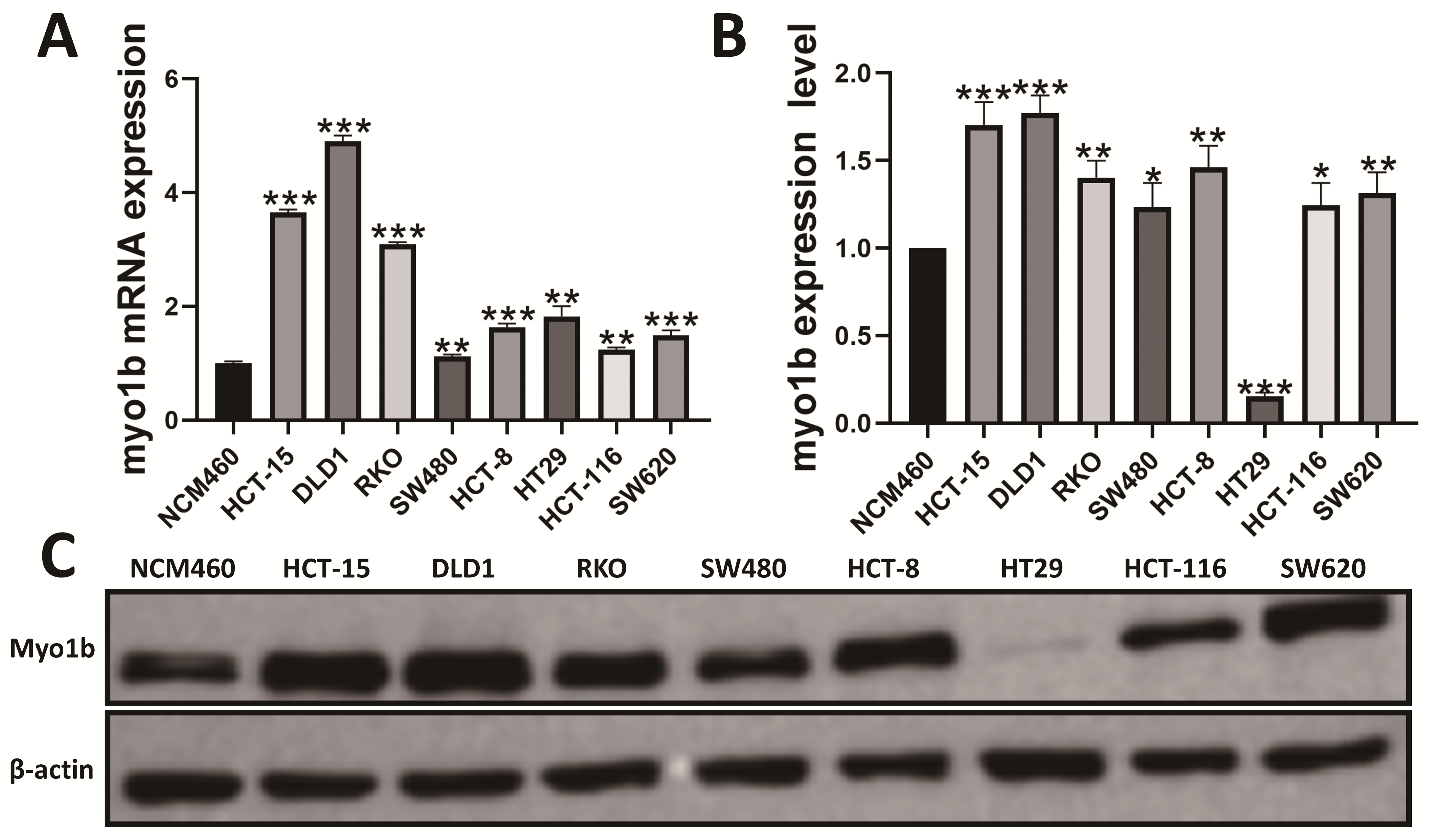


**Supplementary Figure S1 (A) & (B) & (C)**Expression of Myo1b mRNA (**A**) and protein (**B & C**) were detected in NCM460 and eight different CRC cell lines. The bar graph **(B)** shows the relative Myo1b expression in CRC cell lines (normalized to β-actin). Each bar represented the mean ± SD (n≥3). *, p < 0.05; **, p < 0.05; ***, p < 0.001.

**Supplementary Figure S2 (A) & (B)** Cell Counting Kit-8 assays **(A)** and Colony Formation assays **(B)** were conducted to evaluate the effect of Myo1b knockdown on CRC cells proliferation. Each bar represented the mean ± SD (n≥3). **(C) & (D) & (E) & (F)** Transwell invasion assay **(C)** and Transwell migration assay **(D)** and wound healing assay **(E) & (F)** were performed to detect the effect of Myo1b knockdown on CRC cells migration and invasion ability. Each bar represented the mean ± SD (n≥3).

**Abbreviations:** SD, standard deviation; *, P < 0.05; **, P < 0.01.


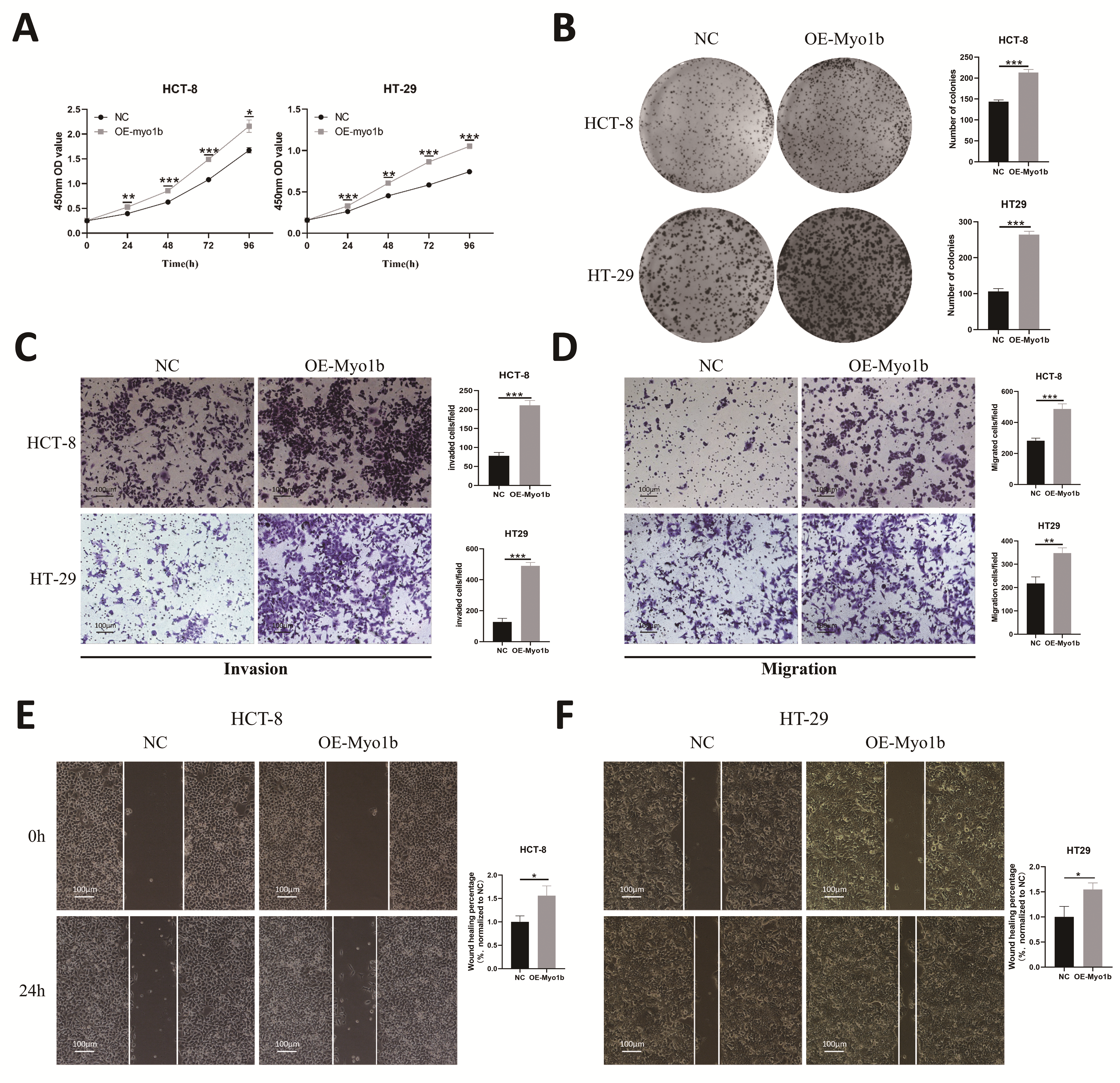


**Supplementary Figure S3 (A) & (B)** Cell Counting Kit-8 assays **(A)** and Colony Formation assays **(B)** were conducted to evaluate the effect of Myo1b overexpression on CRC cells proliferation. Each bar represented the mean ± SD (n≥3). **(C) & (D) & (E) & (F)** Transwell invasion assay **(C)** and Transwell migration assay **(D)** and wound healing assay **(E) & (F)** were performed to detect the effect of Myo1b overexpression on CRC cells migration and invasion ability. Each bar represented the mean ± SD (n≥3).

**Abbreviations:** SD, standard deviation; *, P < 0.05; **, P < 0.01; ***, p < 0.001.

**
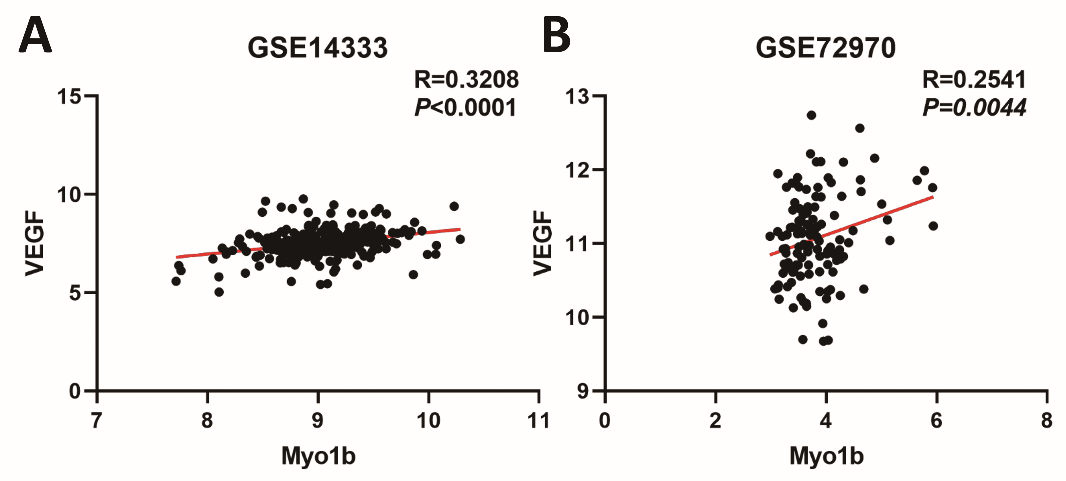
**

**Supplementary Figure S4 (A) & (B)** Pearson correlation analysis was conducted to analyze the relation between Myo1b and VEGF in CRC GEO datasets (GSE14333 (**A**), GSE72970 (**B**)).


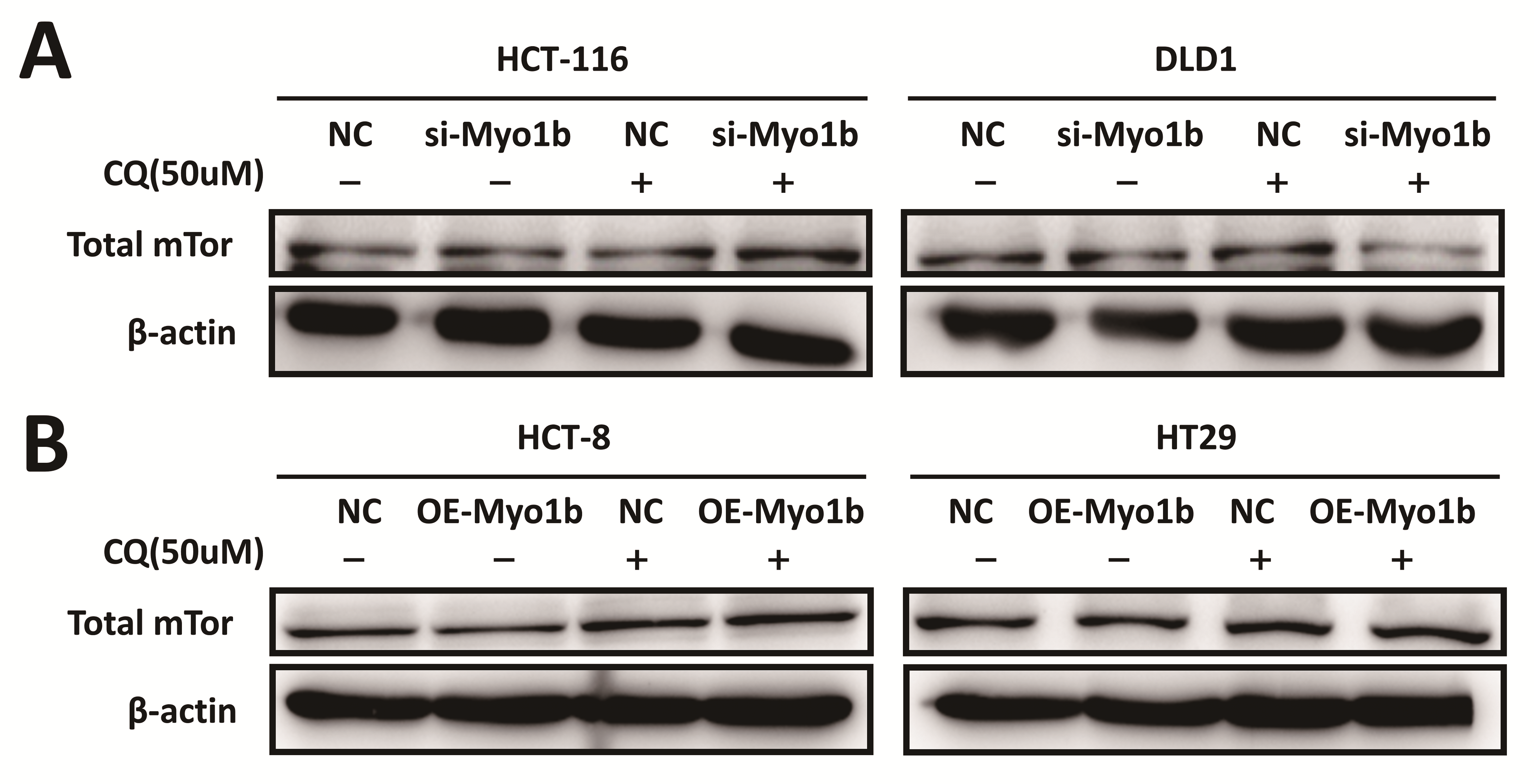


**Supplementary Figure S5 (A) & (B)** Western blot analyses of total mTOR (unphosphorylated protein) in the CRC cells silencing Myo1b **(A)** or overexpressing **(B)** were performed in the presence or absence of Chloroquine (50 μM, 6 h).


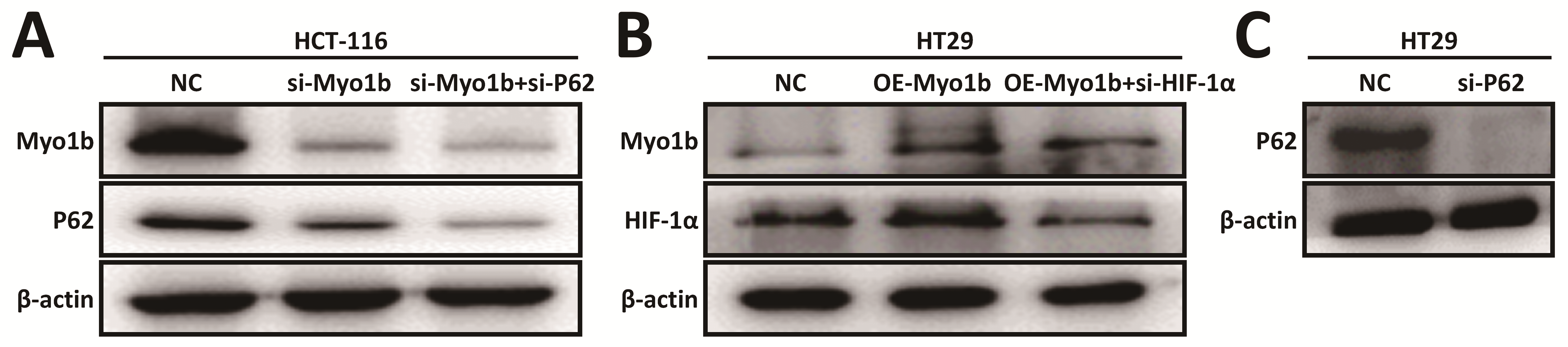


**Supplementary Figure S6 (A) & (B) & (C)** Western blot was conducted to detect the efficiency of si-P62 in HCT-116-si-Myo1b cells **(A)** and si-HIF-1a in HT-29-OE-Myo1b cells **(B)** and si-P62 in HT-29 cells **(C).**
