# Supplementary material for: Myo1b promotes tumor progression and angiogenesis by inhibiting autophagic degradation of HIF-1α in colorectal cancer
Source: Cell Death Dis. 2022 Nov 8;13(11):939. doi: 10.1038/s41419-022-05397-1 (PMC9643372; doi:10.1038/s41419-022-05397-1)
Supplement: Supplementary file 2 — supplemental table [file 41419_2022_5397_MOESM2_ESM.docx]

**Supplementary Table S1.** RT-qPCR primer sequences for human genes

| **Gene** | **Forward primer** | **Reverse primer** |
| --- | --- | --- |
| Myo1b | GCTCTGGTGTGGAGGTCCTA | CGTTGCTTCCTCAGGTCTTC |
| GAPDH | GGAGCGAGATCCCTCCAAAAT | GGCTGTTGTCATACTTCTCATGG |
| Ang-2 | AACTTTCGGAAGAGCATGGAC | CGAGTCATCGTATTCGAGCGG |
| bFGF1 | TTCACAGCCCTGACCGAGAA | CGTTGCTACAGTAGAGGAGTTTG |
| DLL4 | TTAAGCACTTCCAGGCGGTC | GATGAGCGAGAAGGTACCCG |
| IL-8 | TTTTGCCAAGGAGTGCTAAAGA | AACCCTCTGCACCCAGTTTTC |
| PDGF-B | CTCGATCCGCTCCTTTGATGA | CGTTGGTGCGGTCTATGAG |
| PLGF | AGTGGGCCTTGTCTGCTG | CAGCAGGGAGACACAGGATG |
| VEGF-A | AGGGCAGAATCATCACGAAGT | AGGGTCTCGATTGGATGGCA |
| HIF-1a | GAACGTCGAAAAGAAAAGTCTCG | CCTTATCAAGATGCGAACTCACA |

| **siRNA** | **Sequences (5'-3')** |
| --- | --- |
| si-Myo1b | CUUGUUGAUUUCCAGGUAAGC  GCUUACCUGGAAAUCAACAAG |
| si-P62 | GCAUUGAAGUUGAUAUCGAUU  AAUCGAUAUCAACUUCAAUGC |
| NC | UUCUCCGAACGUGUCACGU  ACGUGACACGUUCGGAGAA |

**Supplementary Table S2.** Sequences for siRNAs.

**Supplementary Table S3.** Correlation of subject characteristics and Myo1b expression among colorectal patients.

| **Characteristics** | **Total [n]** | **Myo1b** | |  |
| --- | --- | --- | --- | --- |
|  |  | **Low** | **high** | ***P* value^1^** |
| Tissues |  |  |  | 0.000005 |
| Colorectal cancer | 28 | 11 | 17 |  |
| colonic mucosa | 28 | 28 | 0 |  |
| Sex |  |  |  | 0.685990 |
| Female | 8 | 2 | 6 |  |
| Male | 20 | 9 | 11 |  |
| Age |  |  |  | 0.705080 |
| ≤60 | 13 | 5 | 8 |  |
| ＞60 | 15 | 6 | 9 |  |
| Tumor depth |  |  |  | 0.365217 |
| T2 | 6 | 4 | 2 |  |
| T3+T4 | 20 | 7 | 13 |  |
| Lymph node |  |  |  | 0.475697 |
| - | 15 | 7 | 8 |  |
| + | 13 | 4 | 9 |  |
| Distant metastasis |  |  |  | 0.384541 |
| M0 | 20 | 10 | 10 |  |
| M1 | 7 | 1 | 6 |  |
| AJCC stage |  |  |  | 1 |
| Low | 14 | 6 | 8 |  |
| High | 14 | 5 | 9 |  |
| AJCC stage |  |  |  | 0.481657 |
| 1 | 6 | 4 | 2 |  |
| 2 | 8 | 2 | 6 |  |
| 3 | 7 | 4 | 3 |  |
| 4 | 7 | 1 | 6 |  |

Pearson χ^2^ test of independence between clinicopathological features in colorectal cancer and Myo1b expression.

**Supplementary Table S4.** Correlation of subject characteristics and Myo1b expression among colorectal patients in GSE39084.

| **Characteristics** | **Total [n]** | **Myo1b** | |  |
| --- | --- | --- | --- | --- |
|  |  | **Low** | **high** | ***P* value** |
| TNM stage |  |  |  | 0.006 |
| 1 | 8 | 7 | 1 |  |
| 2 | 23 | 13 | 10 |  |
| 3 | 16 | 3 | 13 |  |
| 4 | 22 | 8 | 14 |  |
| T stage |  |  |  | 0.250 |
| T1+T2 | 12 | 8 | 4 |  |
| T3 | 37 | 15 | 22 |  |
| T4 | 20 | 8 | 12 |  |
| M stage |  |  |  | 0.442 |
| M0 | 48 | 23 | 25 |  |
| M1 | 22 | 8 | 14 |  |
| N stage |  |  |  | 0.026 |
| N0 | 35 | 21 | 14 |  |
| N1 | 20 | 5 | 15 |  |
| N2 | 15 | 5 | 10 |  |
| Braf gene mutation status |  |  |  | 0.287 |
| Mutant | 8 | 2 | 6 |  |
| WT | 62 | 29 | 33 |  |
| Kras gene mutation status |  |  |  | 0.728 |
| Mutant | 30 | 14 | 16 |  |
| WT | 40 | 17 | 23 |  |

Pearson χ^2^ or Fisher’s exact test of independence between clinicopathological features in colorectal cancer and Myo1b expression.
