# Supplementary material for: Myo1b promotes tumor progression and angiogenesis by inhibiting autophagic degradation of HIF-1α in colorectal cancer
Source: Cell Death Dis. 2022 Nov 8;13(11):939. doi: 10.1038/s41419-022-05397-1 (PMC9643372; doi:10.1038/s41419-022-05397-1)
Supplement: Supplementary file 3 — Full and uncropped western blots [file 41419_2022_5397_MOESM3_ESM.docx]

**Figure 1E**

**
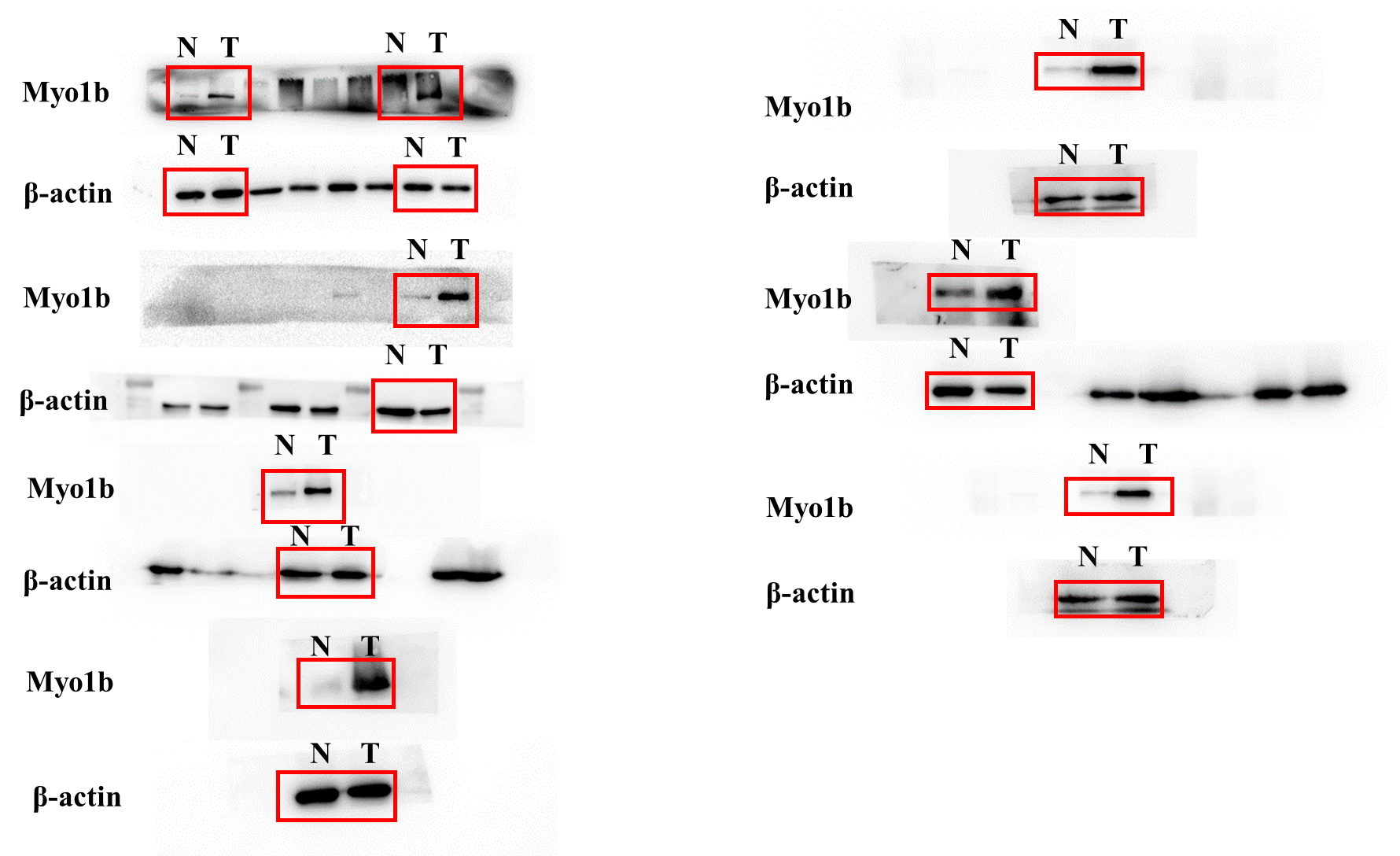
**

**Figure 3B**

**
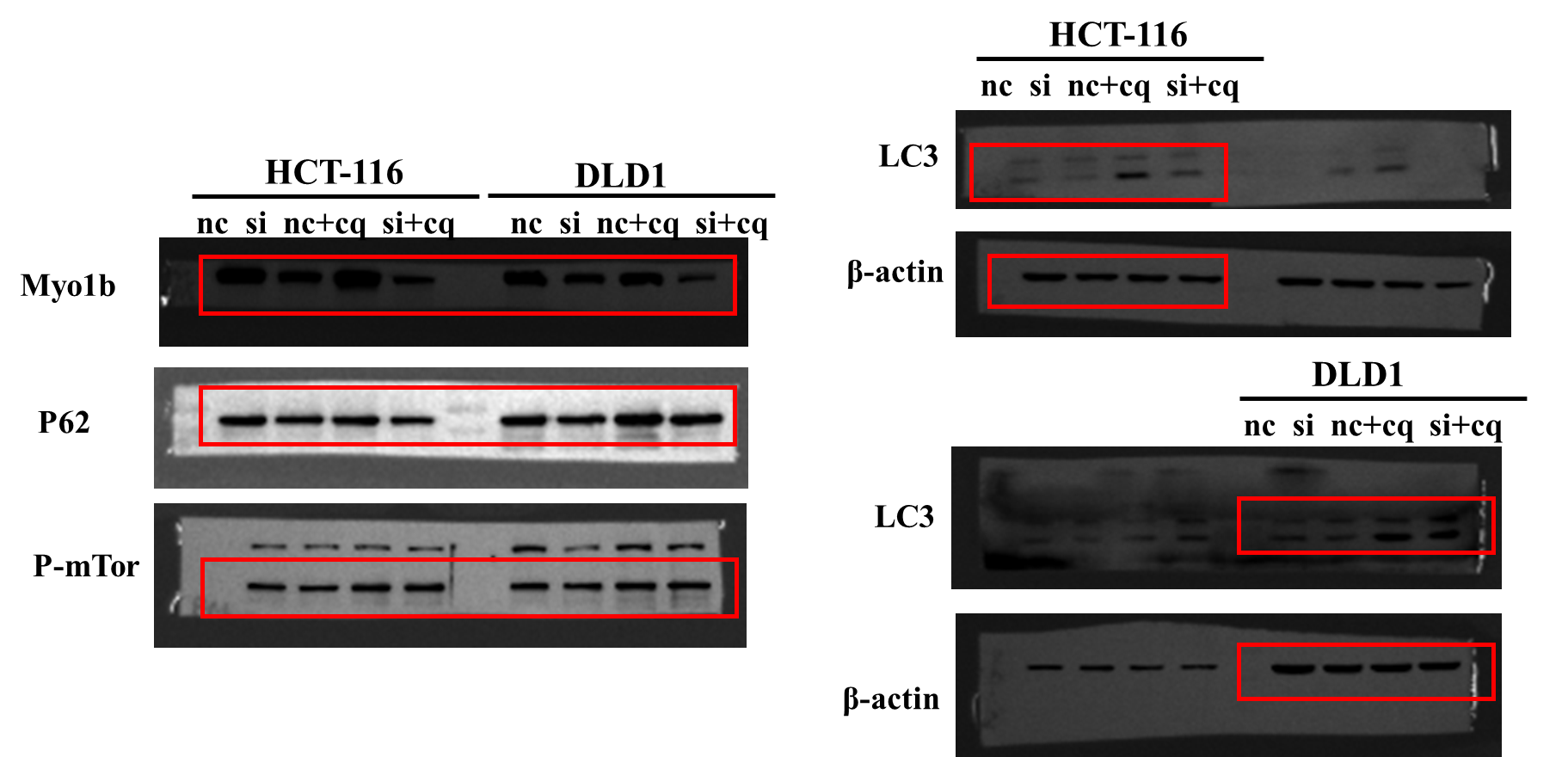
**

**Figure 3C**

**
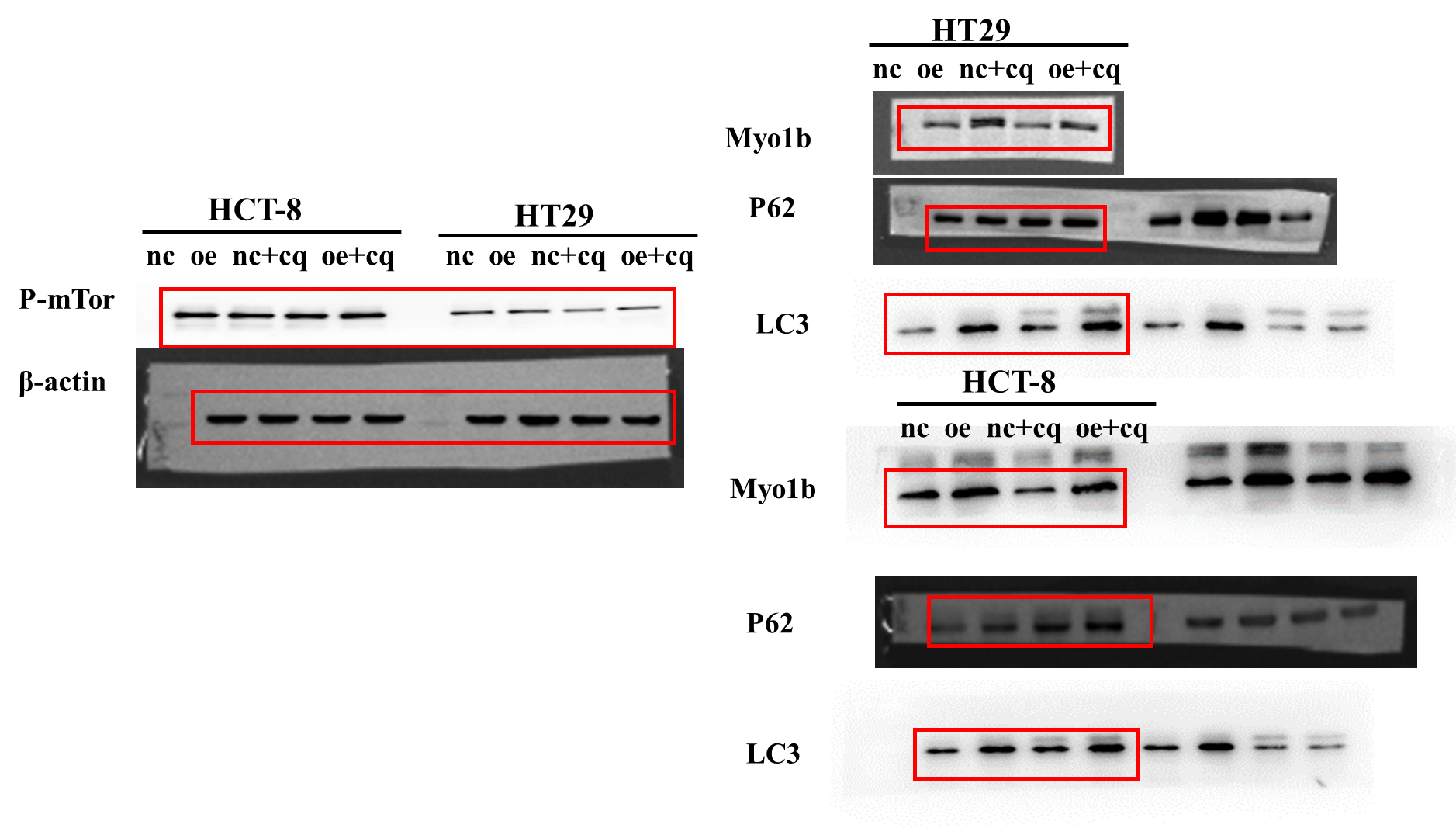
**

**Figure 4A**

**
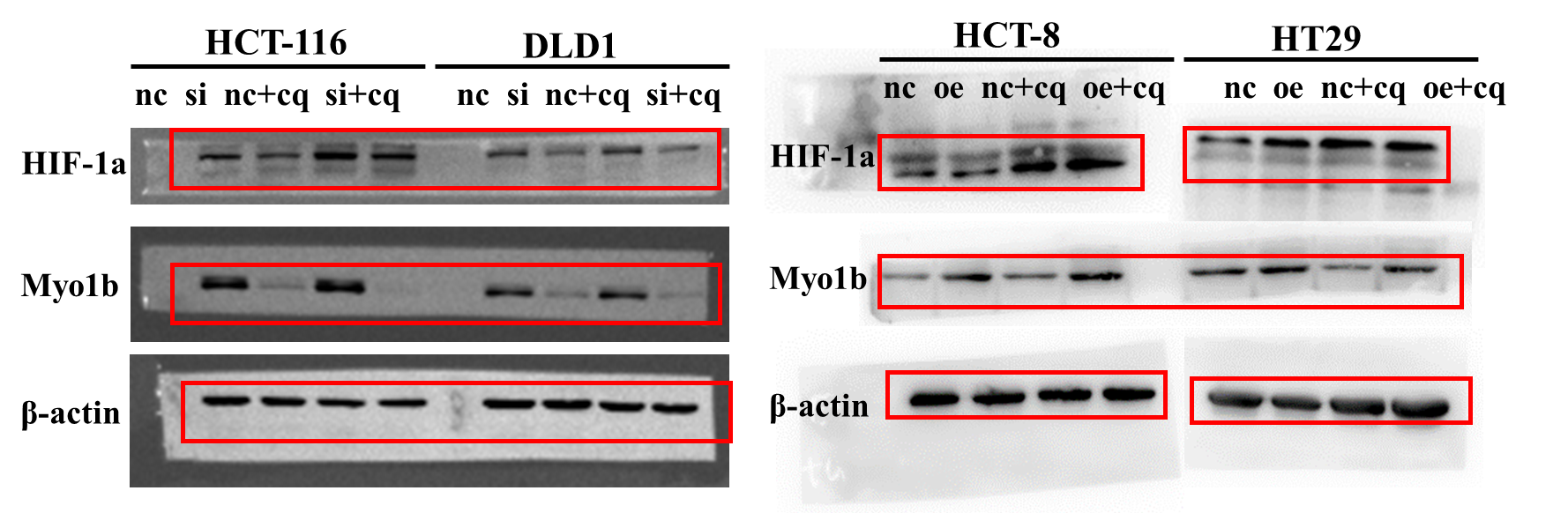
**

**Figure 4C**

**
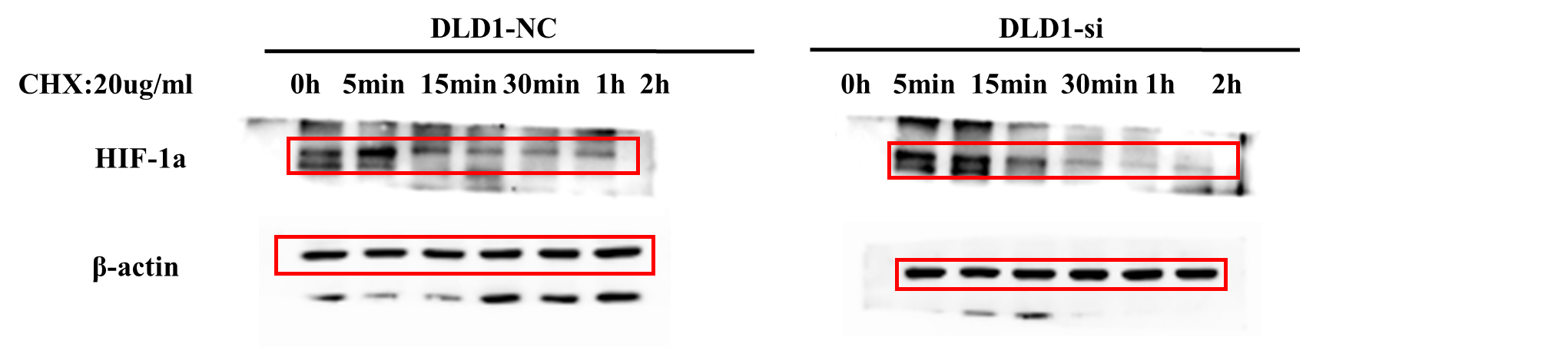
**

**Figure 4D**

**
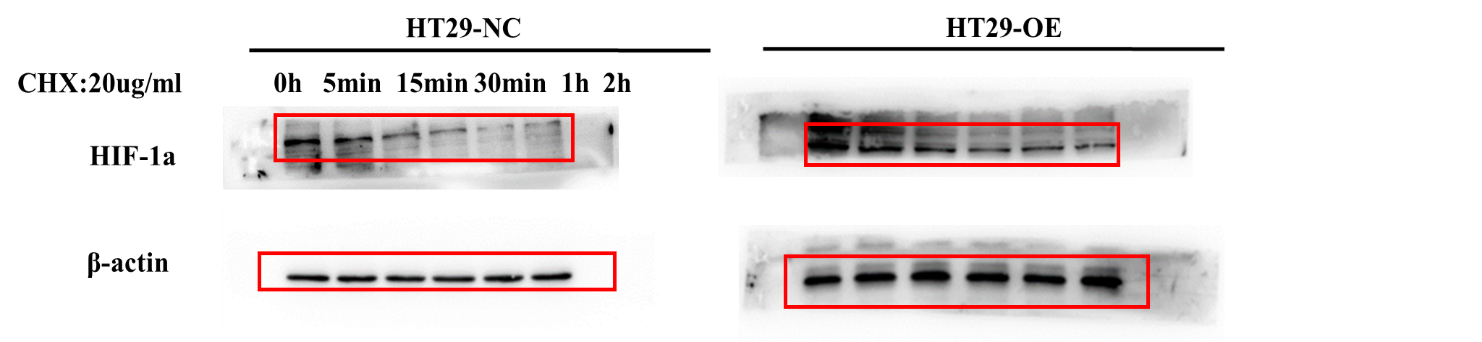
**

**Figure 4E**

**
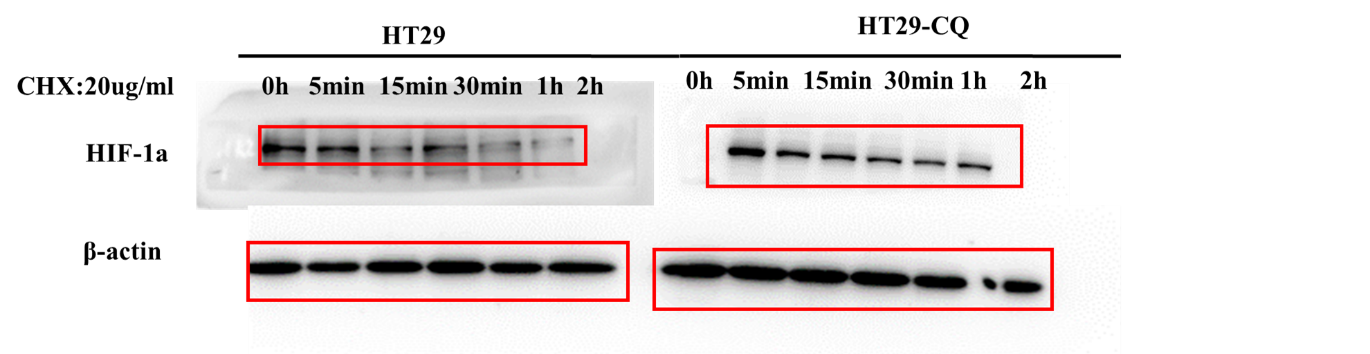
**

**Figure 4F**

**
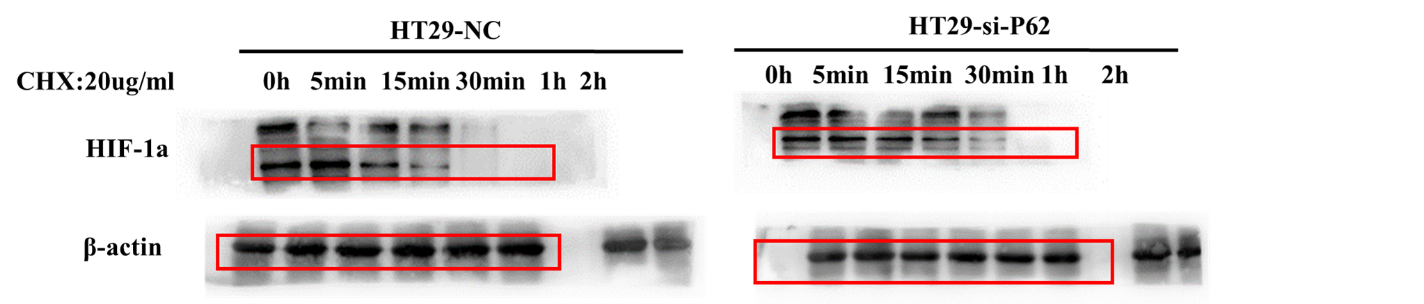
**

**Figure 4G**

**
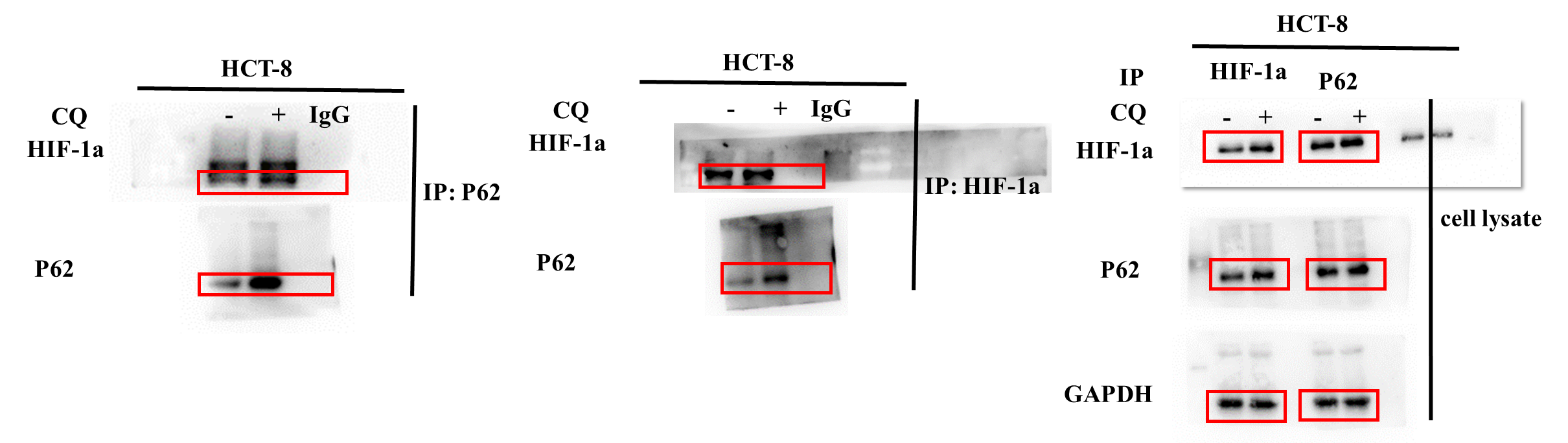
**

**Figure 6A**

**
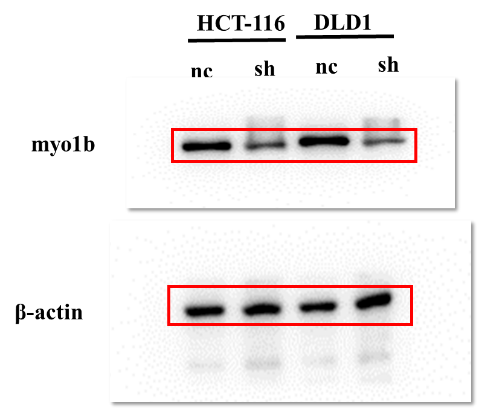
**

**Figure S1B**

**
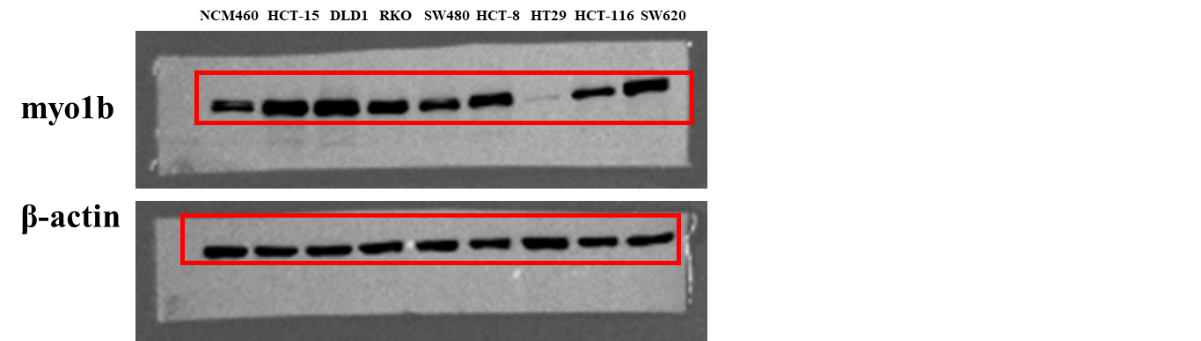
**

**Figure S5A**

**
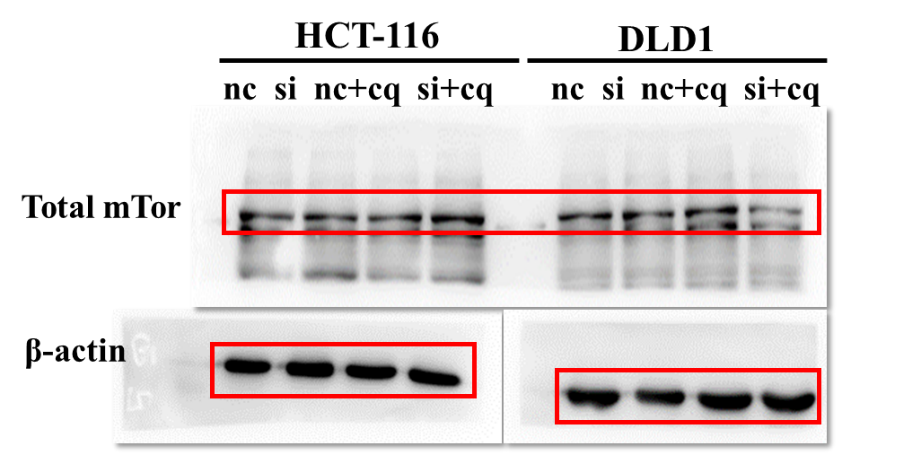
**

**Figure S5B**

**
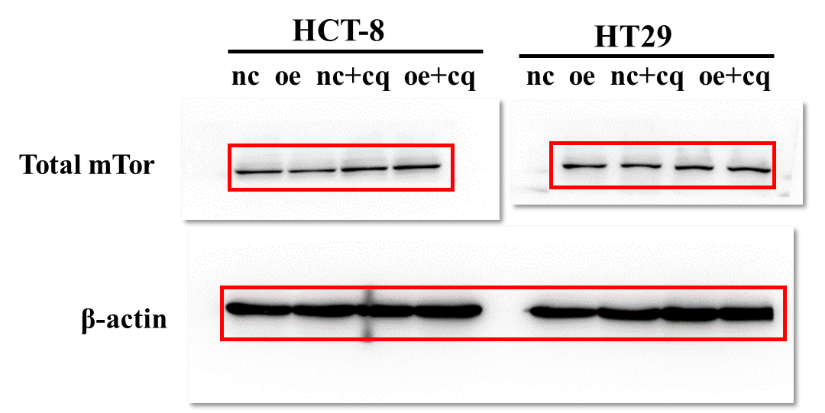
**

**Figure S6A**

**
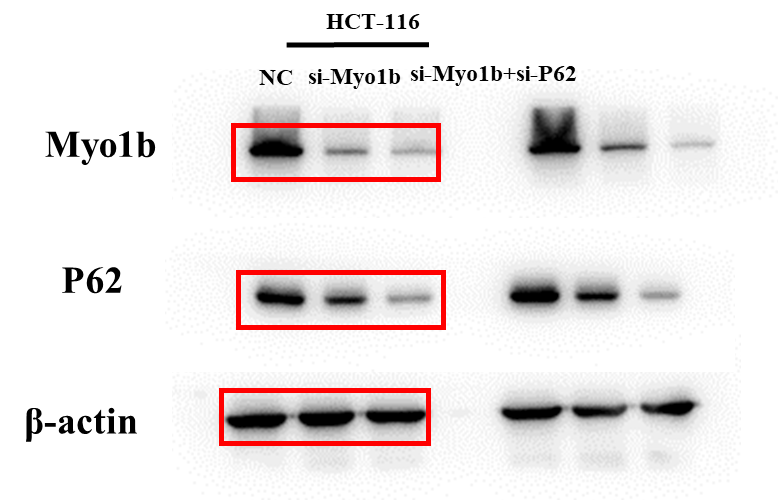
**

**Figure S6B**

**
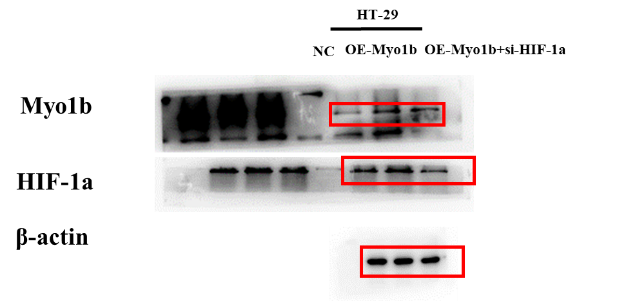
**

**Figure S6C**

**
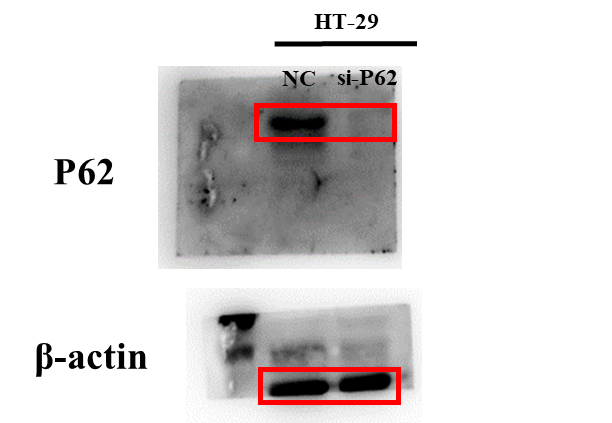
**
